# Protein Tyrosine Phosphatase 1B‐Mediated Granulosa Cell Insulin Resistance Links Metabolic Stress to Aging‐Relevant Ovarian Dysfunction and Is Reversed by Gengnianchun

**DOI:** 10.1111/acel.70583

**Published:** 2026-06-09

**Authors:** Yanqiu Rao, Ting Xu, Yan Ding, Jun Li, Lingyun Gao, Yun Wang, Wenjun Wang

**Affiliations:** ^1^ Department of Integrated Western and Traditional Medicine Obstetrics and Gynecology Hospital of Fudan University Shanghai China; ^2^ Shanghai Key Laboratory of Reproduction and Development, Shanghai Key Laboratory of Female Reproductive Endocrine‐Related Diseases Obstetrics & Gynecology Hospital of Fudan University Shanghai China; ^3^ International Peace Maternity and Child Health Hospital, Shanghai Jiao Tong University Shanghai China

**Keywords:** cellular senescence, Chinese herbal medicine, granulosa cells, insulin resistance, ovarian aging, protein tyrosine phosphatase 1B

## Abstract

Metabolic disorders, particularly insulin resistance, are increasingly recognized as accelerators of female reproductive decline. However, the molecular mechanisms by which peripheral metabolic stress translates into impaired ovarian reserve remain incompletely understood. Here, we propose that protein tyrosine phosphatase 1B (PTP1B), a negative regulator of insulin signaling, serves as a molecular bridge linking systemic insulin resistance to aging‐relevant ovarian dysfunction and can be pharmacologically targeted. By integrating transcriptomic profiling of granulosa cells from women with diminished ovarian reserve (DOR) with network‐based pharmacology and IR‐DOR‐associated gene annotations, we identified PTP1B as a candidate mediator of a metabolic stress‐associated ovarian dysfunction axis. In a high‐fat diet (HFD)‐induced mouse model of systemic insulin resistance and metabolic stress‐associated ovarian dysfunction, treatment with the traditional multi‐herbal formula Gengnianchun (GNC) improved systemic glucose homeostasis, restored estrous cyclicity, and preserved primordial and growing follicles. These effects were accompanied by reduced ovarian PTP1B expression, reactivation of IRS1–AKT2 signaling, and enhanced GLUT4‐mediated glucose handling in granulosa cells. In human granulosa‐like KGN cells, GNC selectively restored insulin signaling and cell migration under insulin‐resistant conditions; these effects were phenocopied by PTP1B knockdown and attenuated by PTP1B overexpression or pharmacological inhibition of AKT2. Collectively, these findings identify ovarian PTP1B as a key mediator of metabolic stress‐associated, aging‐relevant ovarian dysfunction and highlight PTP1B‐directed interventions, including GNC, as potential strategies to preserve ovarian function in metabolically vulnerable states.

## Graphical abstract

Metabolic stress induces insulin resistance and upregulates PTP1B in granulosa cells, leading to aging‐relevant ovarian dysfunction. Gengnianchun (GNC) suppresses PTP1B, restores IRS1–AKT2–GLUT4 signaling, and preserves follicles, highlighting PTP1B as a therapeutic target for ovarian dysfunction in metabolically vulnerable states.

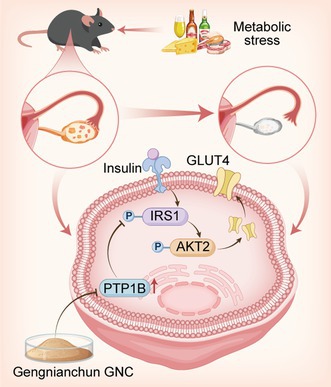

**Abbreviations**
AFC: antral follicle count
AKT2: AKT serine/threonine kinase 2
AMH: anti‐Müllerian hormone
DOR: diminished ovarian reserve
E2: estradiol
FSH: follicle‐stimulating hormone
GLUT4: glucose transporter type 4
GNC: Gengnianchun formula
HFD: high‐fat diet
HOMA‐IR: homeostatic model assessment of insulin resistance
IF: immunofluorescence
IHC: immunohistochemistry
IPGTT: intraperitoneal glucose tolerance test
IR: insulin resistance
IRS1: insulin receptor substrate 1
ITT: insulin tolerance test
LH: luteinizing hormone
MET: metformin
mTOR: mechanistic target of rapamycin kinase
PPI: protein–protein interaction
PTP1B/PTPN1: protein tyrosine phosphatase 1B
siRNA: small interfering RNA

## 1 Introduction

Female reproductive aging is characterized by the progressive decline in ovarian reserve and deterioration of oocyte quality (Wang et al. 2023; Wu et al. 2025). Although largely chronologically programmed, this process is profoundly influenced by systemic metabolic health, with insulin resistance (IR) and related metabolic disturbances emerging as potent accelerators of ovarian functional decline (Xue et al. 2024). Clinically, IR has been associated with diminished ovarian reserve (DOR), a condition marked by accelerated follicle depletion and distinct from classical polycystic ovary syndrome (Lu and Xia 2023; Robeva et al. 2024). This association underscores that metabolic stress can serve as a key driver of premature ovarian aging (He et al. 2025). However, the molecular mechanisms linking peripheral insulin insensitivity to ovarian functional decline remain poorly defined.

Within ovarian follicles, granulosa cells are essential for folliculogenesis and steroidogenesis, and their function critically depends on proper glucose metabolism and insulin sensitivity (Tian et al. 2025). Integrative transcriptomic and network‐based analyses of granulosa cells from women with DOR have revealed dysregulated insulin signaling, mitochondrial dysfunction, and stress response pathways, reflecting an accelerated aging phenotype within the ovarian niche (Liu and Fang 2025; Wei et al. 2024). These findings implicate granulosa cell–specific insulin resistance as a potential link between systemic metabolic stress and reproductive senescence (Alberico and Woods 2021; Xu et al. 2019). Identifying the upstream molecular node that disrupts insulin action in these cells is therefore critical to understanding metabolic stress‐associated ovarian dysfunction.

Protein tyrosine phosphatase 1B (PTP1B, encoded by PTPN1) is a well‐established negative regulator of insulin signaling that dephosphorylates the insulin receptor and its substrate IRS1, thereby attenuating downstream PI3K/AKT activity (Liu et al. 2025; Zhang et al. 2016). Beyond its systemic metabolic role, emerging evidence links PTP1B to cellular aging, with elevated activity associated with mitochondrial dysfunction, oxidative stress, and age‐related functional decline (Olloquequi et al. 2022; Yang et al. 2023). Despite this, PTP1B's expression, regulation, and functional relevance in the ovary under metabolic stress remain largely unexplored (Hense et al. 2024). We therefore hypothesized that PTP1B may act as a molecular bridge, translating systemic insulin resistance into aging‐relevant ovarian dysfunction by compromising granulosa cell function.

To experimentally probe this hypothesis, we employed the multi‐herbal formula Gengnianchun (GNC), which has reported efficacy in ameliorating age‐related reproductive disorders (Gao et al. 2020; Zhao and Wang 2020). GNC contains circulating bioactive compounds, including paeoniflorin, berberine, and icariin, which influence metabolic pathways and insulin sensitivity (Li et al. 2018; Rondanelli et al. 2021; Sánchez‐Gutiérrez et al. 2024; Wu et al. 2023). We reasoned that GNC could serve both as a therapeutic intervention and as a pharmacological probe to dissect PTP1B‐mediated pathways in ovarian dysfunction under metabolic stress.

In this study, we combined transcriptomic analysis of granulosa cells from women with DOR with network‐based bioinformatics to nominate PTP1B as a candidate mediator linking metabolic stress to aging‐relevant ovarian dysfunction. We then validated this target in an HFD‐induced mouse model of systemic insulin resistance and in insulin‐resistant human granulosa‐like KGN cells. Mechanistic analyses showed that GNC restored granulosa‐cell insulin signaling and function at least in part through suppression of the PTP1B–IRS1–AKT2–GLUT4 axis. These findings provide insight into how metabolic stress is translated into ovarian dysfunction and highlight PTP1B as a potential therapeutic target for preserving ovarian function in metabolically vulnerable states.

## 2 Material and Methods

### 2.1 Network Pharmacology Analysis and Molecular Docking

#### 2.1.1 Identification of Candidate Drug Targets and Disease‐Associated Genes

Nine major blood‐borne compounds of GNC were selected based on previously published UPLC‐Q‐TOF/MS pharmacokinetic data (Peng et al. 2023; Rao et al. 2025). Putative protein targets were predicted using SwissTargetPrediction ( *Homo sapiens* ; probability > 0.1) and standardized with UniProt identifiers (Coudert et al. 2023).

Disease‐associated genes were compiled from multiple sources. Granulosa cell differentially expressed genes (DEGs) were obtained from the public GEO dataset GSE232306, which served as the discovery cohort. In the original study, 60 women of advanced maternal age were recruited, including 30 with DOR and 30 with normal ovarian reserve (NOR). Six granulosa‐cell samples per group were randomly selected for RNA‐seq analysis. DOR was defined according to the POSEIDON criteria as age 35–42 years with antral follicle count (AFC) < 5 or anti‐Müllerian hormone (AMH) < 1.2 ng/mL (Jia et al. 2023). Because clinical and metabolic indices required to assess insulin resistance were not available in GSE232306, this dataset could not be used to determine the prevalence of clinical insulin resistance among DOR patients. Therefore, GSE232306 was used to define DOR‐associated granulosa‐cell transcriptional changes, while IR‐DOR‐associated genes were independently retrieved from GeneCards and DisGeNET (Piñero et al. 2021; Stelzer et al. 2016). Aging‐related genes were obtained from the Human Aging Genomic Resources (HAGR) database. DEGs were defined as |log_2_FC| > 1 with *p* < 0.05. The overlap among GNC compound targets, GSE232306‐derived DEGs, IR‐DOR‐associated genes, and aging‐related genes was visualized using a Venn diagram.

#### 2.1.2 Protein–Protein Interaction and Compound–Target–Pathway Network Construction

**FIGURE 1:**
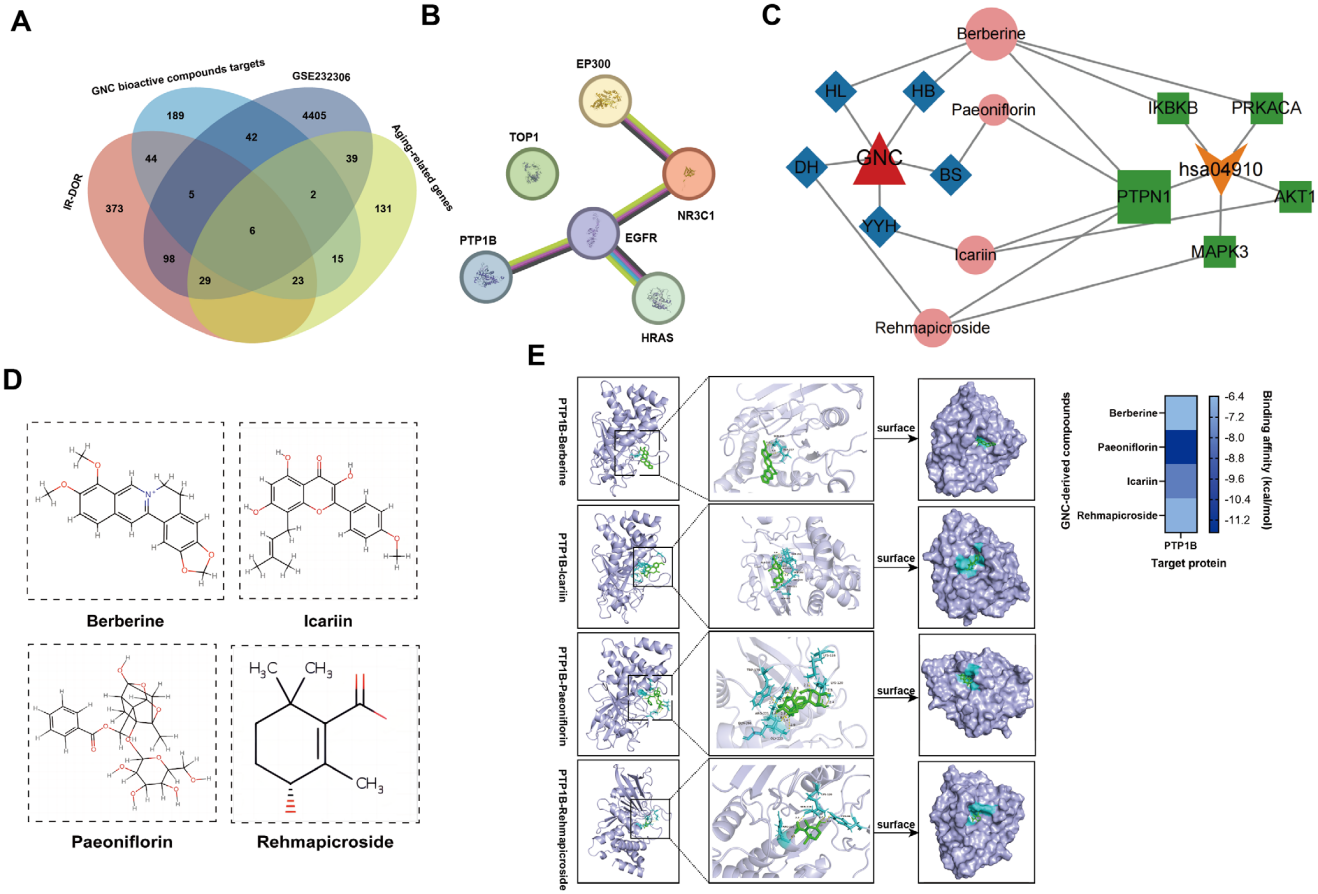
Network pharmacology and transcriptomic screening identify PTP1B as a potential target of GNC in insulin resistance‐associated ovarian aging. (A) Venn diagram showing the overlap among GNC bioactive‐compound targets, GSE232306‐derived granulosa‐cell DEGs, IR‐DOR‐associated genes retrieved from GeneCards and DisGeNET, and HAGR aging‐related genes. (B) PPI network of the six overlapping candidate genes. (C) Compound–target–pathway network linking representative GNC compounds with predicted targets and the KEGG insulin signaling pathway. PTPN1 encodes PTP1B. (D) Chemical structures of berberine, icariin, paeoniflorin, and rehmapicroside. (E) Molecular docking of the four compounds with PTP1B. Binding energies are shown in kcal/mol.

To prioritize candidate mediators for experimental validation, the six overlapping genes identified in the Venn analysis (Figure 1A) were analyzed using the STRING database (v11.5, confidence score ≥ 0.7) to construct a protein–protein interaction (PPI) network. The network was visualized in Cytoscape (v3.9.1) to assess predicted functional connectivity among candidate genes.

A compound–target–pathway network was constructed in Cytoscape by linking bioactive GNC compounds to their predicted targets and mapping these targets to the insulin signaling pathway (KEGG map hsa04910). Node shapes and colors were assigned as follows: red triangle for the GNC formula, blue diamonds for herb sources, pink circles for plasma‐detectable bioactive compounds, green squares for target genes, and an orange triangle for the KEGG pathway. Edges represent predicted compound–target interactions. Layout adjustments were made to improve clarity and minimize node and edge overlap.

#### 2.1.3 Molecular Docking Validation

Chemical structures of four representative GNC compounds (paeoniflorin, berberine, icariin, and rehmapicroside) were retrieved from PubChem and energy‐minimized using the MMFF94 force field. The three‐dimensional structure of PTP1B (PDB ID: 7MNF) was obtained from the RCSB Protein Data Bank and prepared by removing water molecules and adding polar hydrogens. Site‐directed docking was performed using AutoDock Vina (v1.2.0), focusing on the catalytic pocket (grid center: *x* = 30.519, *y* = 13.047, *z* = 40.049; grid size: 47 × 39 × 42 points; spacing: 0.375 Å) (Torgeson et al. 2022). The lowest‐energy binding pose for each compound was analyzed using PyMOL v2.5. Binding energies ≤ −7.0 kcal/mol were considered suggestive of favorable predicted binding and were used for comparative prioritization rather than as direct evidence of physical interaction.

### 2.2 Preparation and Quality Control of GNC

GNC granules were obtained from Jiangyin Tianjiang Pharmaceutical Co. Ltd. (Batch no. 1910304; 3.8 g per sachet) and comprised 12 herbal components (Table 1). All in vivo and in vitro experiments used the same qualified batch to ensure within‐study consistency.

**TABLE 1:** Composition of Gengnianchun formula.

Chinese name	Accepted scientific name	Family	Plant part	Crude herb (g)
Shudihuang	*Rehmannia glutinosa (Gaertn.) Libosch. ex Fisch. & C.A.Mey*.	Orobanchaceae	Root, prepared	15
Yinyanghuo	*Epimedium acuminatum* Franch.	Berberidaceae	Herba/Leaf (aerial parts)	12
Baishao	*Paeonia lactiflora* Pall.	Paeoniaceae	Root	12
Gouqizi	*Lycium barbarum* L.	Solanaceae	Fructus (dried fruit)	12
Guiban	*Plastrum testudinis (animal drug; plastron)*	Testudines	Plastron	15
Zhimu	*Anemarrhena asphodeloides* Bunge	Asparagaceae	Rhizome	15
Tusizi	*Cuscuta australis* R.Br.	Convolvulaceae	Seed	12
Bajitian	*Morinda officinalis* F.C.How	Rubiaceae	Root	12
Congrong	*Cistanche deserticola* Y.C.Ma	Orobanchaceae	Stem	12
Huangbai	*Phellodendron chinense* C.K.Schneid.	Rutaceae	Rhizome	9
Huanglian	*Coptis chinensis* Franch.	Ranunculaceae	Rhizome	3
Fuling	*Wolfiporia cocos (F.A.Wolf) Ryvarden & Gilb. (syn. Poria cocos)*	Polyporaceae (Fungi)	Sclerotium	9

Batch qualification was carried out by HPLC‐DAD using paeoniflorin, berberine, and icariin as representative marker compounds. The reported concentration ranges were derived from same‐batch analytical determinations under different extraction conditions rather than from inter‐batch comparisons. Representative chromatograms and quantitative results are provided in Figure S1 and Table S1, respectively. The contents of paeoniflorin, berberine, and icariin were 0.35%–0.44%, 0.31%–0.39%, and 0.09%–0.11%, respectively, and met the predefined quality specifications for GNC granules.

To support assessment of systemic exposure after oral administration, previously generated plasma pharmacochemistry data were considered, and paeoniflorin was additionally quantified in plasma from female Sprague–Dawley rats following repeated GNC administration. The pharmacokinetic parameters of paeoniflorin are summarized in Table S2. GNC was therefore treated as a standardized multi‐herb formula rather than as a preparation represented by any single constituent. Accordingly, the in vitro experiments were performed using GNC‐containing serum after oral administration of the complete formula rather than direct exposure to paeoniflorin alone.

### 2.3 In Vivo Animal Studies and GNC Intervention

#### 2.3.1 Animals, Experimental Design, and Drug Administration

All animal procedures were approved by the Institutional Animal Care and Use Committee of Fudan University (Approval No. 2024‐FCKYY‐084) and were performed in accordance with institutional guidelines. Female C57BL/6J mice (6–8 weeks old; Shanghai SLAC Laboratory Animal Co. Ltd.) were housed under specific pathogen‐free (SPF) conditions with a 12 h light/dark cycle, controlled temperature (20°C ± 2°C), and humidity (60% ± 5%).

The in vivo experiments consisted of a preliminary validation cohort and a therapeutic intervention cohort. The preliminary cohort was used to confirm that 6 weeks of 45% kcal HFD feeding induced systemic insulin resistance and ovarian dysfunction before therapeutic testing; this cohort was not included in treatment efficacy analyses.

For the intervention study, mice were acclimated for 1 week and then randomly assigned to four groups (*n* = 8 per group): Control, HFD, HFD+GNC, and HFD+MET. Control mice were maintained on standard chow, whereas mice in the HFD groups were fed a 45% kcal fat diet (D12451, Research Diets) for 6 weeks to establish metabolic stress‐associated ovarian dysfunction. HFD‐fed mice continued on the same diet and received daily oral gavage of vehicle, GNC (1.4 g/kg/day), or metformin (200 mg/kg/day) for an additional 6 weeks. Metformin was included as an insulin‐sensitizing pharmacological positive control. The control group defined baseline metabolic and ovarian parameters; the HFD group established the metabolic stress‐associated ovarian dysfunction phenotype; and the two intervention groups were used to determine whether GNC could reverse an established metabolic stress phenotype relative to vehicle and metformin. The 6‐week HFD induction period was selected on the basis of published evidence and a previously validated model showing early insulin resistance and ovarian dysfunction without severe obesity‐related confounding (Lang et al. 2019). GNC and metformin doses were selected based on prior dose‐ranging and rodent intervention studies (Gao et al. 2023; Peng et al. 2023). Treatments and outcome assessments were performed by investigators blinded to group allocation. Basic animal characteristics are summarized in Table S3.

#### 2.3.2 Metabolic and Reproductive Phenotyping

Body weight was recorded weekly. Intraperitoneal glucose tolerance tests (IPGTT; 2 g/kg) were performed after a 16 h fast, and insulin tolerance tests (ITT; 0.75 U/kg) were performed after a 4 h fast. Blood glucose was measured from the tail vein at the indicated time points using a handheld glucometer (Accu‐Chek; Roche Diagnostics GmbH, Mannheim, Germany) (Xu et al. 2023).

Fasting serum insulin was measured by ELISA (mLbio, Cat# mL001983). Insulin resistance was assessed using multiple complementary readouts, including fasting insulin, Homeostatic Model Assessment of Insulin Resistance (HOMA‐IR), IPGTT, and ITT. HOMA‐IR was calculated as fasting glucose (mmol/L) × fasting insulin (mIU/L)/22.5, according to the original homeostasis model assessment (Matthews et al. 1985).

Estrous cyclicity was assessed by daily vaginal cytology during the final 3 weeks of treatment. Serum follicle‐stimulating hormone (FSH; CUSABIO, Cat# CSB‐E06871m), luteinizing hormone (LH; CUSABIO, Cat# CSB‐E12770m), estradiol (E_2_; CUSABIO, Cat# CSB‐E07280m), and anti‐Müllerian hormone (AMH; CUSABIO, Cat# CSB‐E13156m) were measured by ELISA according to the manufacturers' instructions.

#### 2.3.3 Ovarian Histomorphological and Molecular Analysis

Ovaries were fixed in 4% paraformaldehyde, paraffin‐embedded, and serially sectioned at 5 μm. Every fifth section was stained with hematoxylin and eosin (H&E) for follicle counting using established morphological criteria. Follicles were classified as primordial, primary, secondary, or antral.

For immunohistochemistry (IHC) and immunofluorescence (IF), sections underwent antigen retrieval and were incubated overnight at 4°C with primary antibodies against PTP1B (Proteintech, Cat# 11334‐1‐AP, 1:100), phospho‐AKT2 (Ser474) (Cell Signaling Technology, Cat# 8599, 1:200), and GLUT4 (Proteintech, Cat# 66846‐1‐Ig, 1:200), followed by appropriate HRP‐conjugated secondary antibodies for IHC (anti‐rabbit IgG, Sigma‐Aldrich, Cat# 12‐348, 1:1000; anti‐mouse IgG, Sigma‐Aldrich, Cat# AP308P, 1:1000) or fluorophore‐conjugated secondary antibodies for IF (anti‐mouse IgG, Sigma‐Aldrich, Cat# SAB4600013, 1:300; anti‐rabbit IgG, Sigma‐Aldrich, Cat# SAB4600107, 1:300). Negative controls without primary antibody were included. Images were acquired under identical exposure settings using a light microscope or confocal microscope (Leica TCS SP8) and quantified with ImageJ.

### 2.4 Mechanistic Studies of GNC in Human Granulosa‐Like KGN Cells In Vitro

#### 2.4.1 Cell Culture and Induction of Insulin Resistance

The human ovarian granulosa‐like tumor cell line KGN was purchased from Cellcook Biotech Co. Ltd. (Guangzhou, China) and cultured in DMEM/F‐12 medium (Gibco, Cat# 11320033) supplemented with 10% fetal bovine serum (FBS, Gibco, Cat# 10099141) and 1% penicillin–streptomycin (Gibco, Cat# 15140122) at 37°C in a humidified 5% CO_2_ incubator.

To establish a mechanistic in vitro system for testing PTP1B‐associated insulin‐signaling defects, logarithmically growing KGN cells were exposed to increasing concentrations of human recombinant insulin (Sigma‐Aldrich, Cat# I2643, 10–640 nM) for 48 h to generate an insulin‐resistant granulosa‐like cell model (KGN‐IR). On the basis of dose–response analysis of glucose consumption, 160 nM insulin was selected for subsequent model induction (Figure S4). The insulin‐resistant phenotype was further validated by acute stimulation with 50 nM insulin, which produced a blunted glucose consumption response in insulin‐pretreated cells compared with naïve KGN cells. This model was then used to determine whether GNC‐containing serum restores insulin responsiveness and whether the PTP1B–IRS1–AKT2 axis is required for this effect.

#### 2.4.2 Preparation of GNC‐Containing Serum

GNC‐containing serum was prepared as previously described with modifications. Adult female Sprague–Dawley rats (6–8 weeks old) were bilaterally ovariectomized to minimize fluctuations in endogenous sex hormones and then randomly administered GNC granules (23.94 g/kg, twice daily) or an equal volume of normal saline by oral gavage for 5 consecutive days. Blood was collected 1 h after the final administration. Serum was separated by centrifugation, heat‐inactivated at 56°C for 30 min, filtered through a 0.22 μm membrane, aliquoted, and stored at −80°C until use.

To determine the working concentration of GNC‐containing serum, KGN cells were exposed to increasing concentrations for 48 h. Cell viability was assessed using the Cell Counting Kit‐8 (CCK‐8; Sigma‐Aldrich, Cat# 96992) according to the manufacturer's instructions. Viability was normalized to untreated controls and plotted on a log_2_ (v/v) concentration scale to reflect the serial dilution design. No apparent cytotoxicity was observed within the tested range, and 10% (v/v) GNC‐containing serum was selected for subsequent experiments on the basis of the dose–response curve and preliminary efficacy observations (Figure S2).

#### 2.4.3 Cell Functional Assays

Glucose consumption was measured using a glucose assay kit (Abcam, Cat# ab65333). After treatment, culture supernatants were collected, and residual glucose concentrations were measured with a microplate reader according to the manufacturer's protocol.

Cell migratory capacity was evaluated using 24‐well Transwell chambers with 8 μm pore membranes (Corning, Cat# 3422). Cells were seeded into the upper chamber in serum‐free medium, and medium containing 10% FBS was added to the lower chamber as a chemoattractant. After 24 h, cells that migrated to the lower membrane surface were fixed, stained with crystal violet (Beyotime, Cat# C0121), and counted in multiple randomly selected microscopic fields.

#### 2.4.4 PTP1B Enzyme Activity Assay

To determine whether GNC‐containing serum directly inhibits PTP1B enzymatic activity, a cell‐free colorimetric phosphatase assay was performed using recombinant human PTP1B (R&D Systems, Cat# 1367‐PT) and p‐nitrophenyl phosphate (pNPP; Sigma‐Aldrich, Cat# P4744) as the substrate. Recombinant human PTP1B was prepared in 50 mM HEPES buffer (pH 7.0) containing 1 mM EDTA and 1 mM DTT. In a 96‐well plate, recombinant PTP1B, pNPP substrate, and serial dilutions of GNC‐containing serum (2%, 4%, 8%, 16%, 32%, and 64% v/v) were mixed in reaction buffer. Control wells received an equal volume of buffer or control serum instead of the test sample. After incubation at 37°C for 30 min, the reaction was terminated by adding 0.1 M NaOH, and absorbance at 405 nm was measured using a microplate reader (BioTek, Synergy H1). Enzymatic activity was normalized to the control group, and the half‐maximal inhibitory concentration (IC50) was estimated by nonlinear regression analysis using GraphPad Prism with a four‐parameter logistic model.

#### 2.4.5 PTP1B Knockdown and Overexpression

KGN‐IR cells were seeded in 6‐well plates at 2 × 10^5^ cells per well and cultured overnight to 60%–70% confluence. For knockdown experiments, cells were transfected with two independent siRNAs targeting human PTP1B (si‐PTP1B#1: 5′‐GAAGUGGAAGGUGAACAAUTT‐3′; si‐PTP1B#2: 5′‐CCAGAAGUGUCCAUGUUUATT‐3′) or a non‐targeting negative control siRNA (si‐NC; GenePharma, Shanghai, China) using X‐tremeGENE siRNA Transfection Reagent (Roche, Cat# 04476093001). Transfection complexes were prepared in Opti‐MEM I reduced‐serum medium (Invitrogen, Cat# 31985070) and added to cells in antibiotic‐free medium at a final siRNA concentration of 50 nM. For overexpression experiments, KGN‐IR cells were transfected with a human PTP1B expression plasmid (PTP1B‐OE; Sino Biological, Cat# HG10811‐UT) or empty vector control (Vector; Sino Biological, Cat# CV012) using Lipofectamine 3000 Transfection Reagent (Thermo Fisher Scientific, Cat# L3000015). Plasmid DNA was used at 1 μg per well. At 48 h after transfection, cells were harvested to verify knockdown or overexpression efficiency or were subjected to GNC‐containing serum treatment and functional assays as indicated. All transfection experiments were performed in at least three independent biological replicates.

#### 2.4.6 AKT2 Signaling Inhibition

To investigate whether AKT2 mediates the effects of GNC in insulin‐resistant granulosa cells, KGN‐IR cells were treated with the AKT2‐pathway inhibitor CCT128930 (1 μM, Abcam, Cat# ab273533). CCT128930 was dissolved in DMSO (Sigma‐Aldrich, Cat# D8418), and an equal volume of DMSO was added to control wells as vehicle control (final DMSO concentration < 0.1%). The concentration of 1 μM was selected based on previous studies and preliminary optimization experiments showing effective pathway inhibition without obvious cytotoxicity (as assessed by CCK‐8 assay; cell viability > 90%). After the indicated treatments, cells were collected for functional and molecular analyses.

#### 2.4.7 Western Blotting and Immunofluorescence Staining

Total protein was extracted using RIPA buffer (Beyotime, Cat# P0013B) supplemented with protease and phosphatase inhibitors (Roche, Cat# 04693159001). Protein concentration was determined using a BCA protein assay kit (Millipore, Cat# 71285‐M). Equal amounts of protein were separated by SDS‐PAGE and transferred onto PVDF membranes (Millipore, Cat# IPVH00010). Membranes were blocked and incubated overnight at 4°C with primary antibodies against PTP1B (Proteintech, Cat# 11334–1‐AP, 1:1000), IRS1 (Abcam, Cat# ab70538, 1:500), phospho‐IRS1 (Tyr612; Thermo Fisher, Cat# PA5‐104595, 1:500), AKT2 (Abcam, Cat# ab126930, 1:500), phospho‐AKT2 (Ser474) (Cell Signaling Technology, Cat# 8599, 1:500), mTOR (Abcam, Cat# ab114179, 1:500), phospho‐mTOR (Ser2448; Cell Signaling Technology, Cat# 5536, 1:500), GLUT4 (Proteintech, Cat# 66846–1‐Ig, 1:2000), and β‐actin (Proteintech, Cat# 60004‐1‐Ig, 1:5000). Membranes were then incubated with HRP‐conjugated secondary antibodies (Proteintech, Cat# SA00001‐1 and SA00001‐2, 1:5000). Protein bands were visualized using enhanced chemiluminescence reagent (Millipore, Cat# WBKLS0500) and quantified by densitometry using ImageJ. To assess phosphorylation activity, phospho‐IRS1, phospho‐AKT2, and phospho‐mTOR signals were normalized to their corresponding total protein levels, namely total IRS1, total AKT2, and total mTOR, respectively. PTP1B and GLUT4 signals were normalized to β‐actin as a loading control. Total IRS1, AKT2, and mTOR were detected in parallel in the same experimental samples, and all original uncropped Western blot images, including phosphorylated and total protein bands, molecular weight marker ladders, and lane annotations, are provided in the Supporting Information Raw Data.

For immunofluorescence staining, cells were fixed with 4% paraformaldehyde (Beyotime, Cat# P0099), permeabilized with 0.1% Triton X‐100 (Sigma‐Aldrich, Cat# T8787), blocked with 5% bovine serum albumin (Sigma‐Aldrich, Cat# A9418), and incubated overnight at 4°C with primary antibody against GLUT4 (Proteintech, Cat# 66846‐1‐Ig, 1:200), followed by fluorophore‐conjugated secondary antibody (Invitrogen, Cat# A11001, 1:500) and DAPI nuclear counterstaining (Beyotime, Cat# C1005). Images were acquired using a confocal laser scanning microscope (Leica TCS SP8) under identical acquisition settings and analyzed with ImageJ.

### 2.5 Statistical Analysis

Data are presented as mean ± standard deviation (SD). Comparisons between two groups were performed using unpaired two‐tailed Student's *t*‐tests. Comparisons among multiple groups were performed using one‐way ANOVA followed by Tukey's or Sidak's multiple‐comparison test, as appropriate. For experiments involving two independent variables, two‐way ANOVA followed by Sidak's post hoc test was applied. All statistical analyses were performed using GraphPad Prism software (version 9.0).

## 3 Results

### 3.1 Integrative Analysis Prioritizes PTP1B as a Candidate Mediator Linking Insulin Resistance to Aging‐Relevant Ovarian Dysfunction

To identify candidate molecular targets linking GNC to metabolic stress‐associated ovarian dysfunction, we integrated predicted GNC targets, HAGR aging‐related genes, IR‐DOR‐associated genes retrieved from GeneCards and DisGeNET, and GSE232306‐derived granulosa‐cell transcriptomic changes. Nine major blood‐borne compounds of GNC were first defined from prior pharmacochemical analysis, and their putative protein targets were predicted using SwissTargetPrediction and standardized by UniProt annotation. In parallel, granulosa‐cell DEGs were obtained from the public GSE232306 dataset, which compared women with DOR and women with normal ovarian reserve. Intersection of these datasets yielded six overlapping candidate genes: TOP1, EGFR, HRAS, NR3C1, EP300, and PTPN1 (Figure 1A).

To further prioritize these candidates, we constructed a PPI network using STRING. Among the six overlapping genes, PTPN1, which encodes PTP1B, was retained as a candidate of particular interest because of its connected position within the candidate network and its established role as a negative regulator of insulin signaling (Figure 1B). We next constructed a compound‐target‐pathway network linking major plasma‐detectable GNC compounds to insulin signaling‐related targets. Four representative compounds—paeoniflorin, berberine, icariin, and rehmapicroside—were all linked to PTPN1/PTP1B within the network context (Figure 1C), and their chemical structures are shown in Figure 1D. Molecular docking further suggested favorable predicted binding of these compounds to the catalytic pocket of PTP1B, with binding energies ranging from −8.1 to −11.68 kcal/mol (Figure 1E). Among them, paeoniflorin showed the lowest predicted binding energy. Together, these analyses prioritized PTP1B as a candidate mediator linking insulin resistance‐related signaling to aging‐relevant ovarian dysfunction and provided a rationale for experimental validation.

### 3.2 GNC Attenuates HFD‐Induced Metabolic Stress‐Associated Ovarian Dysfunction In Vivo

To validate the prediction that PTP1B is a candidate target of GNC, we first confirmed the HFD‐induced model of systemic insulin resistance and metabolic stress‐associated ovarian dysfunction in a preliminary cohort (Figure S3). After 6 weeks of HFD feeding, mice exhibited increased body weight, impaired glucose tolerance and insulin sensitivity, and elevated fasting insulin and HOMA‐IR (Figure S3A–C), confirming systemic metabolic dysfunction. HFD feeding also disrupted reproductive parameters, including increased serum FSH and LH, decreased E_2_ and AMH, irregular estrous cycles, and reduced ovarian follicle counts (Figure S3D–F), indicating robust induction of ovarian dysfunction under metabolic stress.

**FIGURE 2:**
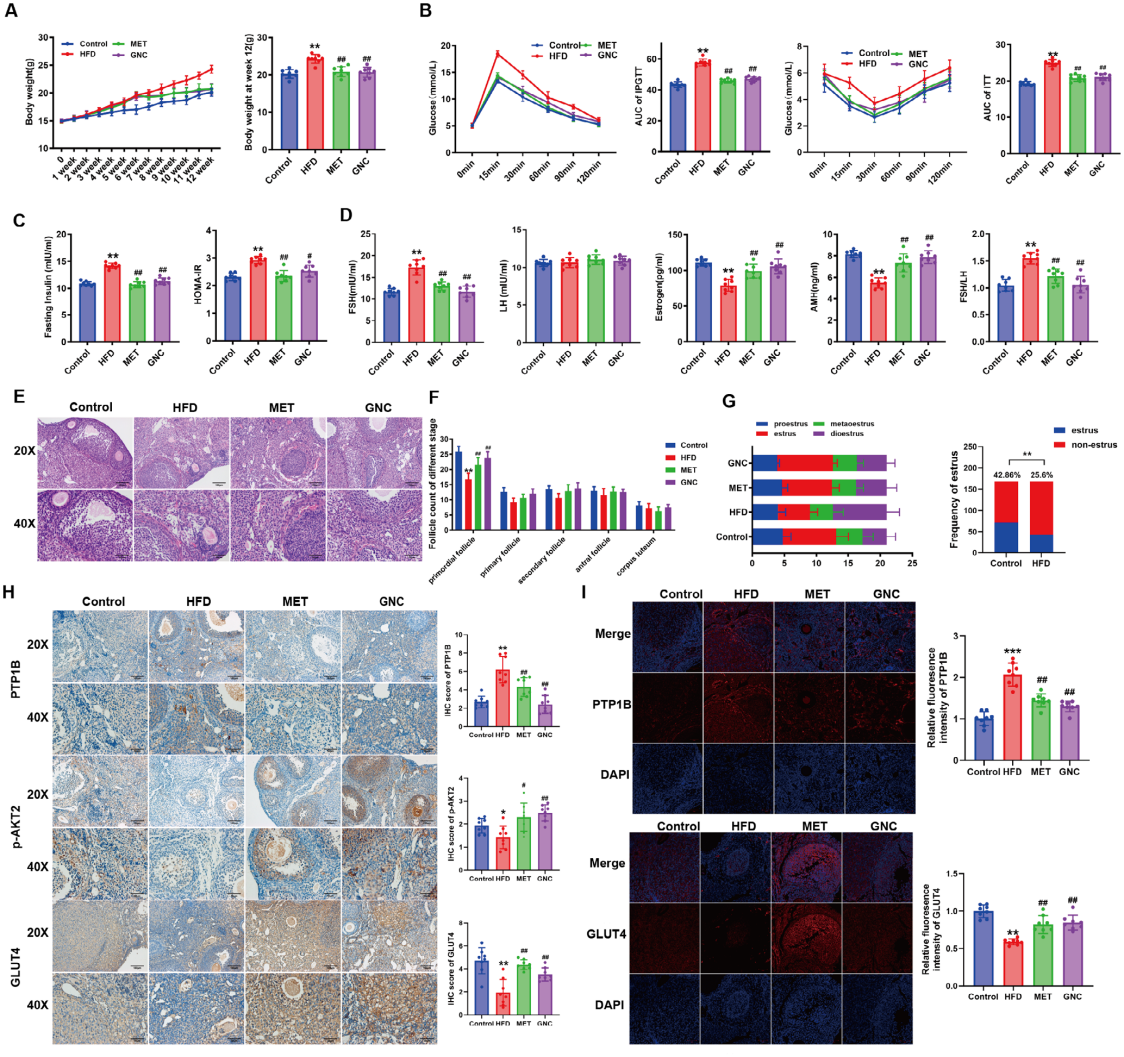
GNC improves HFD‐induced insulin resistance and ovarian dysfunction in mice. Female C57BL/6J mice were fed a HFD for 6 weeks and then treated with vehicle, GNC, or metformin (MET) for 6 weeks. (A) Body‐weight changes during the experimental period and final body weight. (B) IPGTT and ITT curves with corresponding AUC analyses. (C) Fasting serum insulin and HOMA‐IR. (D) Serum levels of FSH, LH, E_2_, AMH, and FSH/LH ratio. (E) Representative ovarian H&E staining. (F) Quantification of follicles at different developmental stages and corpora lutea. (G) Estrous‐cycle stage distribution and estrus/non‐estrus frequency. (H) Representative ovarian IHC staining and IHC scores for PTP1B, p‐AKT2, and GLUT4. (I) Representative IF staining and quantification of PTP1B and GLUT4 fluorescence intensity in ovarian granulosa‐cell regions. Scale bars, 100 μm for 20× images and 50 μm for 40× images in (E, H); 50 μm in (I). Data are mean ± SD; *n* = 8 mice per group. *p < 0.05, **p < 0.01, ***p < 0.001 versus Control; ^#^ *p* < 0.05, ^##^ *p* < 0.01 versus HFD.

In the main cohort, 6 weeks of GNC intervention mitigated these disturbances. HFD‐induced body weight gain was reduced by GNC (Figure 2A), and glucose tolerance and insulin sensitivity were improved, as reflected by decreased area under the curve (AUC) values in IPGTT and ITT analyses (Figure 2B). Fasting insulin levels and HOMA‐IR were also lowered (Figure 2C), indicating amelioration of systemic insulin resistance. We next evaluated reproductive endocrine function. HFD feeding increased serum FSH and the FSH/LH ratio and decreased E_2_ and AMH levels, whereas GNC treatment restored a more favorable hormonal profile, characterized by reduced FSH and FSH/LH ratio together with increased E_2_ and AMH levels (Figure 2D). Histological analysis showed that HFD feeding disrupted ovarian morphology and reduced follicular reserve, whereas GNC treatment improved ovarian architecture and preserved follicles at multiple developmental stages (Figure 2E). Quantitative follicle analysis further confirmed that GNC increased primordial, primary, secondary, and antral follicle numbers, as well as corpus luteum counts, compared with HFD‐fed mice (Figure 2F). Consistent with these histological improvements, GNC also improved reproductive cyclicity, as shown by a more balanced estrous‐stage distribution and increased estrus frequency relative to the HFD group (Figure 2G). At the molecular level, ovarian immunohistochemistry showed that HFD feeding increased PTP1B expression and reduced p‐AKT2 and GLUT4 signals, whereas GNC treatment decreased PTP1B expression and restored p‐AKT2 and GLUT4 levels (Figure 2H). Immunofluorescence analysis further confirmed reduced PTP1B expression and enhanced GLUT4 expression in ovarian granulosa‐cell regions after GNC treatment (Figure 2I). Together, these results show that GNC counteracts HFD‐induced systemic and ovarian dysfunction, and suggest that modulation of the ovarian PTP1B‐AKT2‐GLUT4 axis contributes to its protective effects.

### 3.3 Insulin‐Resistant KGN Cells Recapitulate PTP1B‐Driven Signaling Impairment and Functional Decline

To validate the pathological role of PTP1B in a human‐relevant system, we established an insulin‐resistant model using KGN cells. KGN cells were exposed to increasing concentrations of human recombinant insulin (10–640 nM) for 48 h to determine the optimal model induction condition. Chronic insulin exposure produced a biphasic dose–response in glucose consumption, which peaked at 40 nM and declined at higher concentrations; from 160 nM onward, glucose consumption plateaued at a low level (Figure S4). Based on these data, 160 nM insulin for 48 h was selected as the standard induction condition and was validated in at least three independent experiments.

**FIGURE 3:**
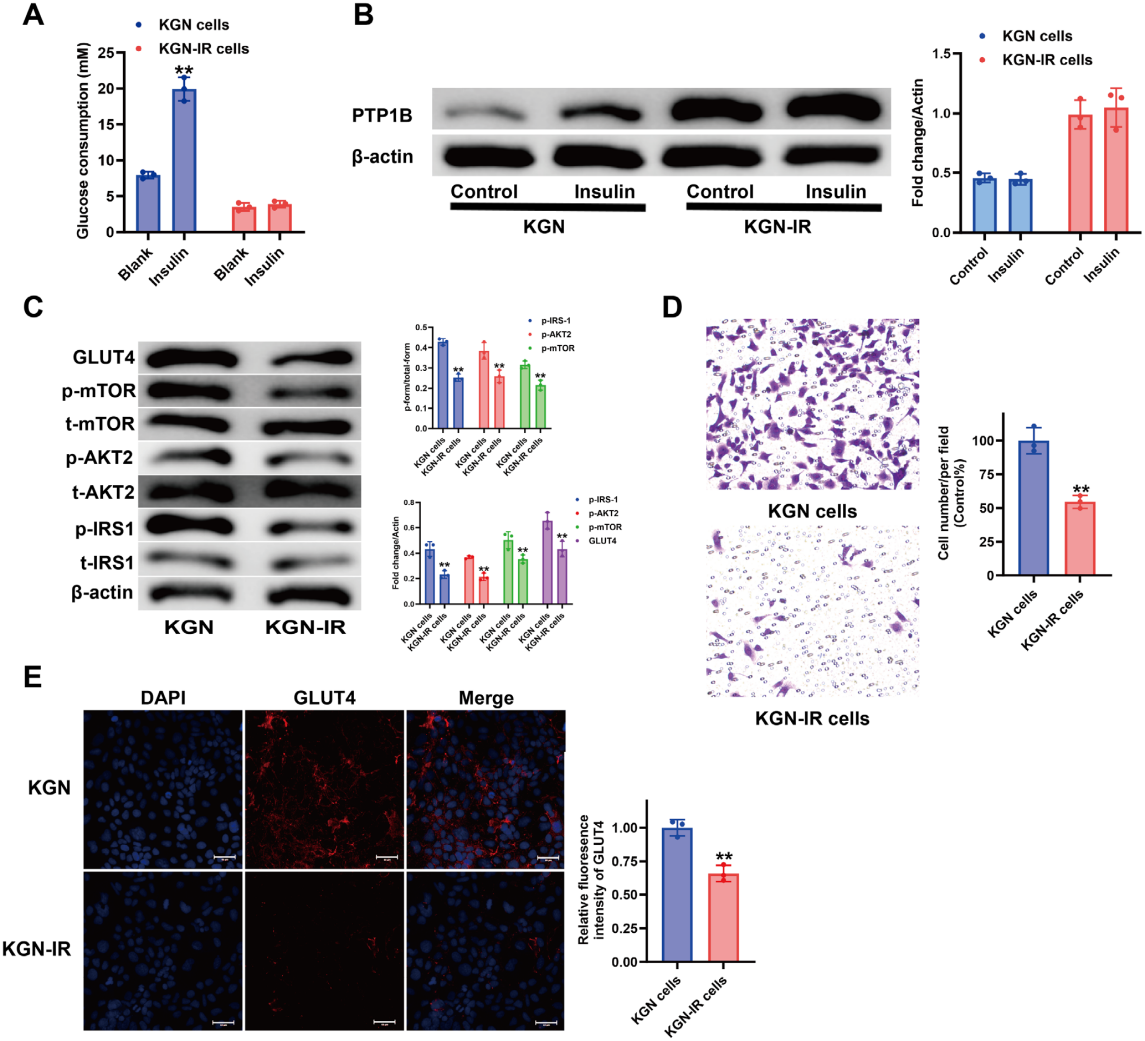
Insulin‐resistant KGN cells display impaired insulin signaling and granulosa‐cell function. KGN‐IR cells were generated by exposure to recombinant insulin at 160 nM for 48 h. (A) Glucose consumption in KGN and KGN‐IR cells after acute insulin stimulation. (B) Representative Western blot and quantification of PTP1B protein expression. (C) Western blot analysis of GLUT4 and IRS1/AKT2/mTOR phosphorylation in KGN and KGN‐IR cells. Phosphorylated IRS1, AKT2, and mTOR were quantified as p/total protein ratios and as p/β‐actin ratios; GLUT4 was normalized to β‐actin. (D) Representative Transwell migration images and quantification of migrated cells. (E) IF staining and quantification of membrane‐associated GLUT4. GLUT4, red; DAPI, blue. Scale bars, 50 μm. Data are mean ± SD from at least three independent experiments. *p < 0.05, **p < 0.01.

Under this condition, KGN‐IR cells exhibited a blunted glucose consumption response to acute insulin stimulation compared with insulin‐sensitive KGN cells, confirming the establishment of an insulin‐resistant phenotype (Figure 3A). Western blot analysis showed that PTP1B protein expression was upregulated in KGN‐IR cells (Figure 3B). Concurrently, insulin signaling was impaired, as indicated by reduced p‐IRS1/IRS1, p‐AKT2/AKT2, and p‐mTOR/mTOR ratios, together with decreased GLUT4 protein expression (Figure 3C). This molecular impairment was accompanied by functional defects. Transwell assays showed markedly reduced migratory capacity in KGN‐IR cells compared with insulin‐sensitive KGN cells (Figure 3D). Immunofluorescence analysis further demonstrated reduced GLUT4 localization at the plasma membrane, reflected by decreased peripheral GLUT4 fluorescence intensity (Figure 3E). Collectively, these results indicate that insulin‐resistant KGN cells exhibit PTP1B upregulation, impaired IRS1–AKT2–mTOR signaling, reduced GLUT4‐associated glucose‐handling capacity, and compromised granulosa‐cell function.

### 3.4 GNC Restores Insulin Sensitivity and Granulosa Cell Function in KGN‐IR Cells

**FIGURE 4:**
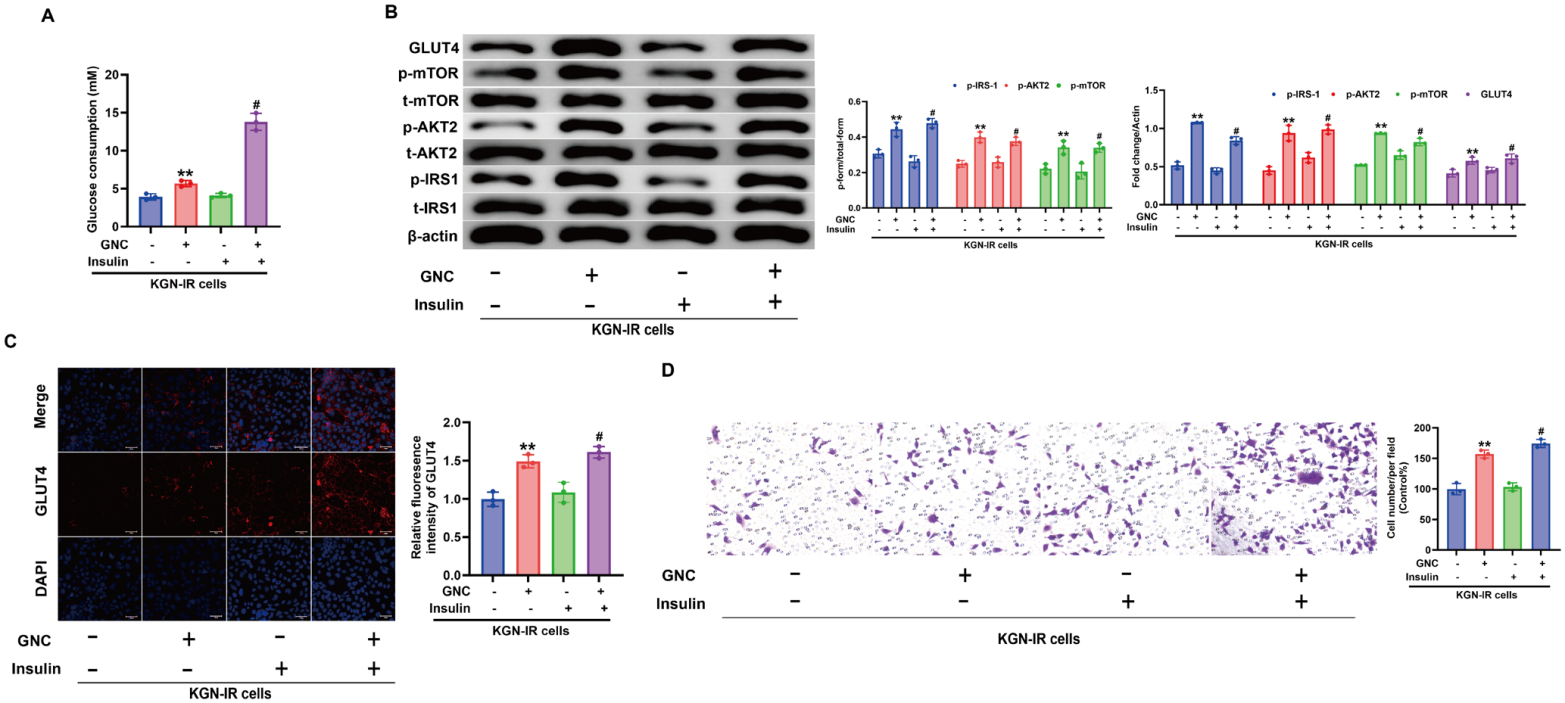
GNC restores insulin responsiveness and cell function in KGN‐IR cells. KGN‐IR cells were treated with control serum or GNC‐containing serum in the absence or presence of insulin. (A) Glucose consumption after the indicated treatments. (B) Western blot analysis of GLUT4 and IRS1/AKT2/mTOR signaling. Phosphorylated IRS1, AKT2, and mTOR were quantified as p/total protein ratios and as p/β‐actin ratios; GLUT4 was normalized to β‐actin. (C) IF staining and quantification of membrane‐associated GLUT4. GLUT4, red; DAPI, blue. (D) Representative Transwell migration images and quantification of migrated cells. Scale bars, 50 μm. Data are mean ± SD from at least three independent experiments. *p < 0.05, **p < 0.01 versus control serum‐treated KGN‐IR cells; ^#^ *p* < 0.05, ^##^ *p* < 0.01 versus insulin‐treated KGN‐IR cells.

To evaluate the therapeutic potential of GNC in vitro, KGN‐IR cells were treated with GNC‐containing serum. Acute insulin alone modestly increased glucose consumption in KGN‐IR cells, whereas co‐treatment with GNC further enhanced this response, indicating partial restoration of insulin sensitivity (Figure 4A). At the molecular level, GNC increased the p‐IRS1/IRS1, p‐AKT2/AKT2, and p‐mTOR/mTOR ratios, together with increased GLUT4 protein expression (Figure 4B). Total IRS1, AKT2, and mTOR were examined in parallel in the original Western blot data and showed no apparent group‐dependent differences. Accordingly, pathway activation was quantified using phospho/total protein ratios for IRS1, AKT2, and mTOR, while GLUT4 expression was normalized to β‐actin. Consistent with these signaling changes, immunofluorescence staining showed enhanced peripheral localization of GLUT4 after GNC treatment (Figure 4C). Transwell assays further demonstrated that GNC significantly improved the impaired migratory capacity of KGN‐IR cells (Figure 4D).

To determine whether the effects were specific to insulin‐resistant cells, we examined insulin‐sensitive KGN cells under basal conditions. In these cells, GNC‐containing serum did not significantly alter glucose consumption, insulin signaling, or GLUT4 localization, whereas insulin stimulation produced the expected enhancement of these responses (Figure S5). These findings indicate that GNC preferentially restores dysregulated insulin signaling and associated functional defects under insulin‐resistant conditions, rather than non‐specifically enhancing basal insulin signaling in insulin‐sensitive granulosa‐like cells.

### 3.5 GNC Restores Insulin Sensitivity and Granulosa Cell Function by Suppressing PTP1B

**FIGURE 5:**
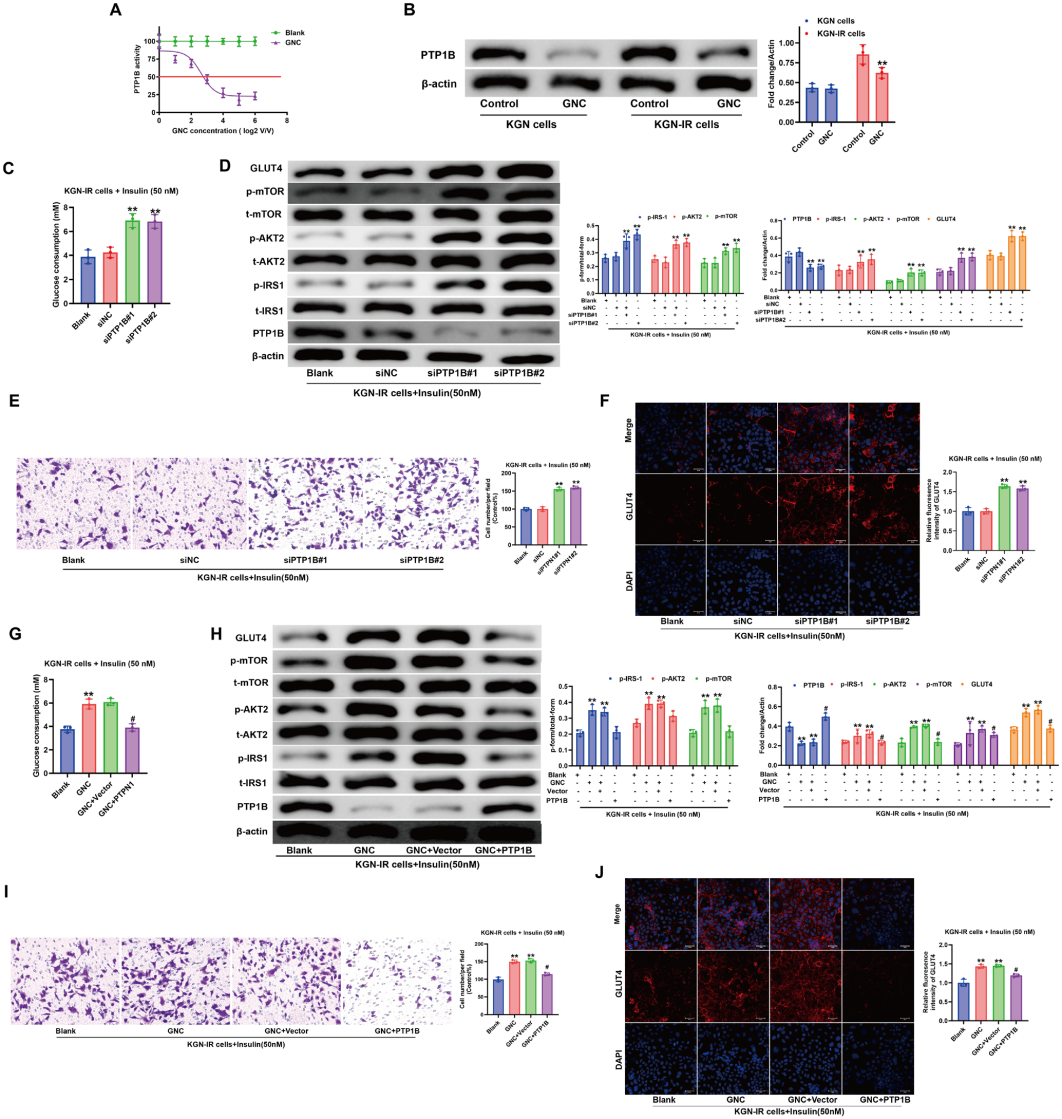
GNC improves insulin sensitivity in KGN‐IR cells by suppressing PTP1B. (A) Dose‐dependent inhibition of recombinant human PTP1B activity by GNC‐containing serum in a cell‐free pNPP assay. (B) Representative western blots and quantification of PTP1B expression in KGN and KGN‐IR cells treated with control serum or GNC‐containing serum. (C) Glucose consumption in insulin‐stimulated KGN‐IR cells after PTP1B knockdown. (D) Western blot analysis of insulin‐signaling proteins after PTP1B knockdown. Phosphorylated IRS1, AKT2, and mTOR were quantified as p/total protein ratios and as p/β‐actin ratios; PTP1B and GLUT4 were normalized to β‐actin. (E) Representative Transwell migration images and quantification after PTP1B knockdown. (F) IF staining and quantification of membrane‐associated GLUT4 after PTP1B knockdown. (G) Glucose consumption in insulin‐stimulated KGN‐IR cells treated with GNC‐containing serum with or without PTP1B overexpression. (H) Western blot analysis of insulin‐signaling proteins after PTP1B overexpression. Phosphorylated IRS1, AKT2, and mTOR were quantified as p/total protein ratios and as p/β‐actin ratios; PTP1B and GLUT4 were normalized to β‐actin. (I) Representative Transwell migration images and quantification after PTP1B overexpression. (J) IF staining and quantification of membrane‐associated GLUT4 after PTP1B overexpression. GLUT4, red; DAPI, blue. Scale bars, 50 μm. Data are mean ± SD from at least three independent experiments; IF quantification was based on 30 cells per group from three independent experiments. *p < 0.05, **p < 0.01 versus the corresponding control; ^#^ *p* < 0.05, ^##^ *p* < 0.01 versus GNC+Vector.

To determine whether GNC‐containing serum can directly modulate PTP1B enzymatic activity, we performed a cell‐free phosphatase assay using recombinant human PTP1B and pNPP as the substrate. GNC‐containing serum inhibited PTP1B activity in a dose‐dependent manner, with nonlinear regression indicating an apparent IC50 of approximately 32% (v/v) (Figure 5A). This result supports the possibility that bioavailable components in GNC‐containing serum can directly suppress PTP1B phosphatase activity. We next examined whether GNC regulates PTP1B expression in KGN‐IR cells. Western blot analysis showed that GNC‐containing serum reduced PTP1B protein expression in insulin‐resistant KGN‐IR cells, whereas insulin‐sensitive KGN cells exhibited no appreciable change (Figure 5B). Thus, the effect of GNC on PTP1B was most evident under insulin‐resistant conditions.

To establish a causal role for PTP1B in mediating the effects of GNC, we applied genetic loss‐of‐function approaches. KGN‐IR cells were transfected with two independent siRNAs targeting PTP1B or a non‐targeting control siRNA. PTP1B knockdown increased glucose consumption compared with the siNC group (Figure 5C). In parallel, PTP1B knockdown restored insulin signaling, as shown by increased p‐IRS1/IRS1, p‐AKT2/AKT2, and p‐mTOR/mTOR ratios, together with increased GLUT4 protein expression (Figure 5D). Functionally, PTP1B knockdown improved granulosa cell behavior, enhancing migratory capacity (Figure 5E) and increasing membrane‐associated GLUT4 signal (Figure 5F). These findings indicate PTP1B suppression is sufficient to phenocopy the restorative effects of GNC in insulin‐resistant granulosa‐like cells.

We then asked whether forced PTP1B overexpression could antagonize the beneficial effects of GNC. In KGN‐IR cells, GNC‐containing serum increased glucose consumption, and this effect was attenuated by PTP1B overexpression but not by vector control (Figure 5G). At the signaling level, GNC‐induced increases in the p‐IRS1/IRS1, p‐AKT2/AKT2, and p‐mTOR/mTOR ratios, together with GLUT4 protein expression, were markedly reduced by PTP1B overexpression (Figure 5H). Consistently, PTP1B overexpression weakened the pro‐migratory effect of GNC (Figure 5I) and diminished GNC‐induced GLUT4 membrane localization (Figure 5J). Taken together, these loss‐ and gain‐of‐function experiments demonstrate that GNC restores insulin sensitivity and granulosa cell function primarily through suppression of PTP1B in the insulin‐resistant granulosa cell context.

### 3.6 AKT2 Activity Is Required for the Beneficial Effects of GNC in KGN‐IR Cells

Having established that GNC alleviates the insulin‐resistant phenotype through suppression of PTP1B, we next asked whether AKT2, a key downstream effector of PTP1B‐regulated insulin signaling, is required for these effects. KGN‐IR cells were pretreated with the AKT2 inhibitor CCT128930 or DMSO vehicle, followed by treatment with control serum or GNC‐containing serum.

**FIGURE 6:**
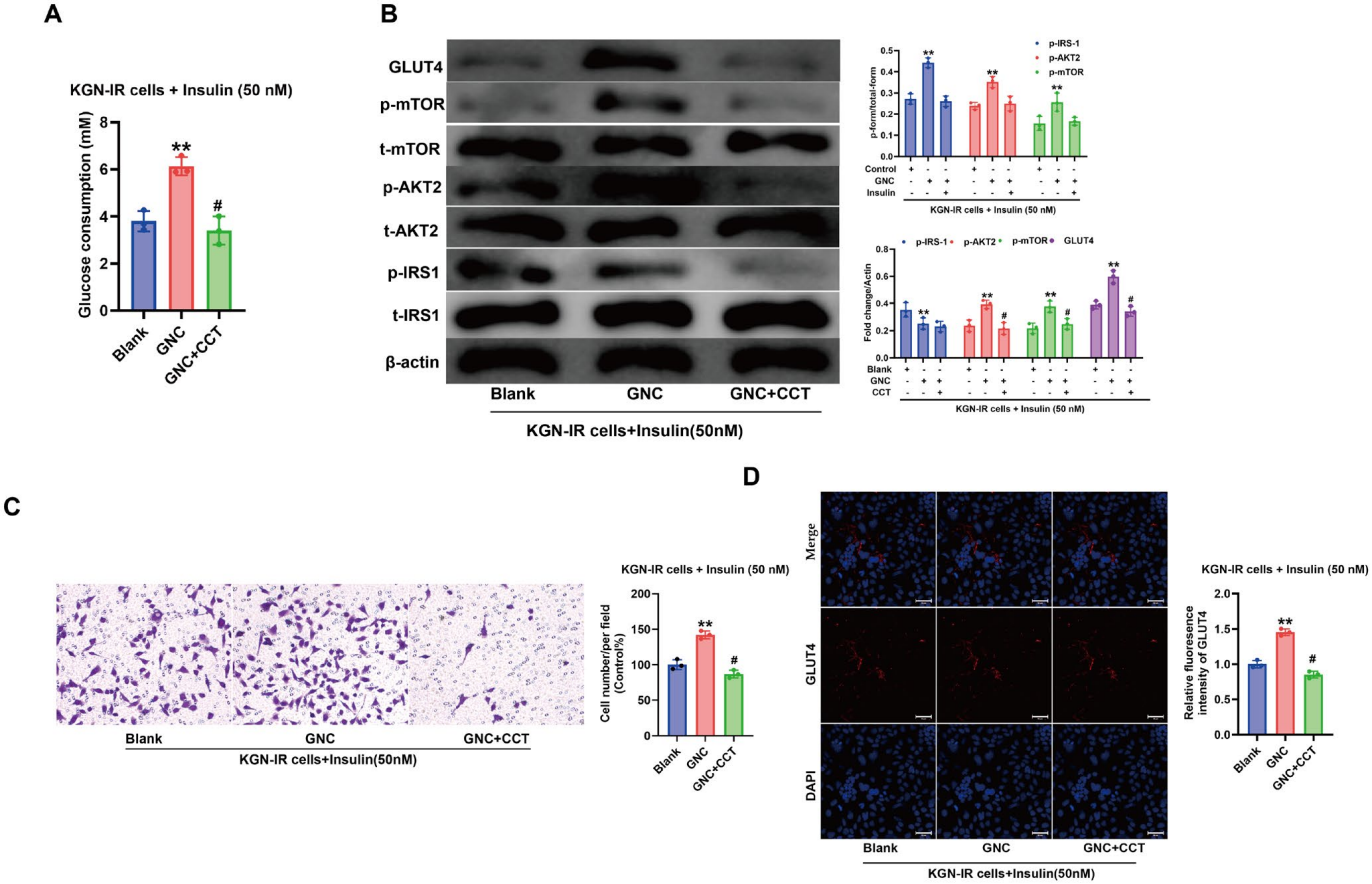
AKT2 activity is required for the beneficial effects of GNC in KGN‐IR cells. KGN‐IR cells were pretreated with the AKT2 inhibitor CCT128930 (CCT) or DMSO vehicle, followed by treatment with control serum or GNC‐containing serum under insulin‐stimulated conditions. (A) Glucose consumption after the indicated treatments. (B) Western blot analysis of GLUT4 and IRS1/AKT2/mTOR signaling. Phosphorylated IRS1, AKT2, and mTOR were quantified as p/total protein ratios and as p/β‐actin ratios; GLUT4 was normalized to β‐actin. (C) Representative Transwell migration images and quantification of migrated cells. (D) IF staining and quantification of membrane‐associated GLUT4. GLUT4, red; DAPI, blue. Scale bars, 50 μm. Data are mean ± SD from at least three independent experiments; biochemical assays included *n* = 6 replicates per group. *p < 0.05, **p < 0.01 versus control serum; ^#^ *p* < 0.05, ^##^ *p* < 0.01 versus GNC‐containing serum.

GNC‐containing serum increased glucose consumption in KGN‐IR cells, whereas this effect was attenuated by CCT128930 (Figure 6A). Western blot analysis showed that GNC increased p‐IRS1/IRS1, p‐AKT2/AKT2, and p‐mTOR/mTOR ratios, together with GLUT4 protein expression, whereas these effects were reduced in the presence of CCT128930 (Figure 6B). Total IRS1, AKT2, and mTOR were detected in parallel in the original Western blot data and showed no apparent group‐dependent changes; therefore, pathway activation was interpreted on the basis of phospho/total protein ratios.

We next assessed whether AKT2 inhibition affected the functional benefits of GNC. Transwell assays showed that GNC‐containing serum enhanced KGN‐IR cell migration, while CCT128930 markedly weakened this effect (Figure 6C). Immunofluorescence analysis further demonstrated that GNC increased membrane‐associated GLUT4 signal in KGN‐IR cells, and this increase was attenuated by CCT128930 treatment (Figure 6D). Collectively, these findings indicate that AKT2 activity is required for the beneficial effects of GNC on glucose metabolism, insulin signaling, cell migration, and GLUT4 redistribution in insulin‐resistant granulosa‐like cells. This supports AKT2 as a critical downstream mediator of the PTP1B‐targeting action of GNC.

## 4 Discussion

The escalating prevalence of metabolic syndrome has highlighted its profound impact on female reproductive longevity, yet the mechanisms by which insulin resistance promotes aging‐relevant ovarian dysfunction remain incompletely defined (He et al. 2025; Tang et al. 2025). Accumulating evidence, including previous work from our group, indicates that granulosa‐cell senescence and metabolic dysfunction are critical cellular events linking systemic insulin resistance to ovarian impairment (Gao et al. 2023). Building on this foundation, the present study identifies PTP1B as an upstream molecular mediator of metabolic stress‐associated granulosa‐cell dysfunction. Our data support a model in which GNC suppresses PTP1B, reactivates the IRS1–AKT2–GLUT4 insulin signaling axis in granulosa cells, and counteracts metabolic stress‐associated ovarian dysfunction. Importantly, the HFD model used here was designed to interrogate metabolically accelerated ovarian dysfunction rather than to fully recapitulate chronological ovarian aging. Thus, our findings define a PTP1B‐centered mechanism operating under metabolic stress, while its relevance to physiological ovarian aging requires validation in naturally aged animals. A notable feature of GNC was its preferential action in metabolically stressed or insulin‐resistant granulosa cells, with limited effects under basal conditions. This context‐dependent restorative profile suggests selective modulation of dysregulated pathways, a desirable property for therapeutic strategies aimed at ovarian dysfunction without disrupting physiological homeostasis.

### 4.1 PTP1B: A Potential Molecular Nexus Linking Metabolic Stress to Aging‐Relevant Ovarian Dysfunction

Our integrated multi‐omics analysis identified PTP1B as a central molecular mediator linking systemic IR to ovarian dysfunction. PTP1B, a negative regulator of insulin and leptin signaling, is well‐established in metabolic disorders such as diabetes and obesity (Delibegović et al. 2024). Beyond these canonical roles, emerging evidence implicates PTP1B in cellular aging via mechanisms involving mitochondrial dysfunction, ER stress, and nutrient‐sensing pathways (Lei et al. 2025). In our study, PTP1B was significantly upregulated under metabolic stress in ovarian tissue, particularly within granulosa cells. Suppression of PTP1B restored insulin signaling and mitigated functional decline, supporting its role as a functional mediator rather than merely a biomarker of metabolic stress‐associated ovarian dysfunction. These findings are reinforced by human genetic data linking metabolic syndrome and ovarian dysfunction, suggesting that PTP1B may serve as a tangible molecular bridge between peripheral metabolic dysregulation and reproductive aging.

Our results also align with the concept of tissue‐specific or “local” insulin resistance as a driver of aging. Analogous examples include myocardial IR contributing to cardiac dysfunction and skeletal muscle IR driving sarcopenia (Caturano et al. 2024). Similarly, granulosa cell–specific IR, potentially mediated by PTP1B upregulation, appears to directly impair folliculogenesis and promote aging‐relevant ovarian dysfunction in addition to systemic metabolic dysfunction.

### 4.2 GNC as a Therapeutic Intervention and Mechanistic Probe

Our in vivo experiments demonstrate that GNC intervention attenuates HFD‐induced metabolic stress‐associated ovarian dysfunction. Treatment with GNC ameliorated systemic insulin resistance, as evidenced by improved glucose tolerance, lower fasting insulin levels, and reduced HOMA‐IR, and concurrently reversed key markers of ovarian dysfunction, including follicular depletion, disrupted hormonal cyclicity, and irregular estrous patterns. These findings provide preclinical validation of clinical observations linking GNC to improved reproductive outcomes in age‐related contexts (Gao et al. 2023), thereby strengthening the biological plausibility of its therapeutic potential.

In mechanistic studies, GNC was leveraged as a pharmacological probe to dissect the underlying pathology. In vitro, GNC‐containing serum selectively restored insulin signaling and functional competence in insulin‐resistant granulosa cells, while exerting minimal effects under normo‐insulinemic conditions. Consistent with its clinical indications, GNC is prescribed for conditions such as perimenopausal syndrome, kidney deficiency, and blood stasis, which are frequently associated with metabolic and reproductive dysfunction, rather than as a tonic for general ovarian enhancement (Zhao and Wang 2020). The observed context‐dependent efficacy aligns with this therapeutic framework, supporting the notion that GNC primarily acts to rectify pathophysiological alterations rather than to non‐specifically amplify basal physiological processes. Collectively, these findings highlight both the mechanistic value and therapeutic relevance of GNC in restoring ovarian function under metabolic stress while preserving physiological homeostasis.

### 4.3 Predictive Insights Into Multi‐Component Synergy Against PTP1B

Our in silico analyses provide a predictive framework for understanding how the multi‐component composition of GNC may collectively target PTP1B. Network pharmacology and molecular docking simulations identified several structurally distinct yet bioavailable compounds within GNC—including paeoniflorin, berberine, icariin, and rehmapicroside—that exhibit potential binding affinity for PTP1B. This finding suggests a polypharmacology strategy, consistent with the emerging paradigm of Chinese herbal formulas, in which multiple bioactive constituents concurrently engage shared targets to produce more robust and balanced effects than single‐agent interventions (Seong et al. 2016). Such a framework provides mechanistic insight into how complex herbal formulations may simultaneously modulate key nodes of pathophysiological signaling—in this case PTP1B—to achieve therapeutic efficacy in metabolically stressed ovarian cells.

To move beyond computational predictions, we performed functional validation using GNC‐containing serum. The complete formulation significantly inhibited PTP1B enzymatic activity in a cell‐free assay, corroborating the docking results and supporting the relevance of the predicted compound‐target interactions. Nevertheless, these findings do not resolve the relative contribution of individual constituents. Future studies employing purified compounds, coupled with quantitative biophysical approaches such as surface plasmon resonance (SPR) and kinetic inhibition assays, will be essential to determine binding affinities, inhibitory potency (IC50), and potential synergistic interactions among the candidate compounds (Zhao et al. 2023). Such analyses will refine our understanding of the mechanistic basis for GNC's multi‐component action and guide rational optimization of therapeutic strategies targeting PTP1B.

### 4.4 The Central PTP1B‐IRS1‐AKT2 Axis and Potential for Complementary Pathways

Our loss‐of‐function and gain‐of‐function studies provide compelling genetic evidence that PTP1B serves as a primary target through which GNC exerts its insulin‐sensitizing effects in granulosa cells. PTP1B knockdown phenocopied the restorative effects of GNC on glucose metabolism, insulin signaling, GLUT4 translocation, and cell migration, whereas PTP1B overexpression attenuated these benefits. Pharmacological inhibition of AKT2 similarly attenuated GNC‐mediated improvements, confirming that reactivation of the AKT2‐GLUT4 axis constitutes a critical downstream event in the mechanism of action (Sharma and Dey 2021).

Despite the centrality of PTP1B‐AKT2 signaling, AKT2 inhibition did not fully abrogate GNC's effects, suggesting the contribution of additional complementary pathways. This observation aligns with the inherent complexity of multi‐herbal formulations, which are expected to act through multiple targets and regulatory networks. For example, previous work demonstrated that GNC can also ameliorate insulin resistance–induced DOR via upregulation of ESR1 and ESR2 within the estrogen signaling pathway (Rao et al. 2025). It is therefore plausible that GNC exerts integrated restorative effects through the coordinated modulation of both the PTP1B‐insulin axis and estrogen signaling pathway, potentially alongside other processes such as oxidative stress regulation and autophagy (Li et al. 2025). Systematic dissection of the individual contributions of GNC components to these parallel pathways remains an important direction for future research to fully elucidate the mechanistic landscape of this multi‐component formula.

### 4.5 Limitations and Future Directions

Several limitations should be considered. First, although the KGN cell line provides a convenient and reproducible model for granulosa cell studies, its tumor origin and divergence from primary cells limit its ability to fully recapitulate the physiology of non‐transformed granulosa cells. Validation in primary granulosa cells from women with insulin resistance and DOR will be critical to enhance translational relevance. Similarly, the HFD‐induced mouse model captures metabolic stress‐associated ovarian dysfunction and aging‐like ovarian changes, but it does not encompass the multifactorial complexity of natural reproductive aging, which involves cumulative oxidative stress, telomere attrition, hormonal shifts, and immune remodeling. Future studies in naturally aged wild‐type female mice will be important to determine whether the PTP1B‐centered mechanism and the protective effects of GNC extend to physiological ovarian aging independent of experimentally induced metabolic stress. In addition, the present in vivo design did not include a normal‐diet + GNC group. Therefore, although our in vitro data suggest that GNC acts preferentially under insulin‐resistant conditions, the current animal study cannot directly determine whether GNC exerts minimal effects in metabolically healthy mice. Future studies including healthy GNC‐treated controls will be required to test the context‐dependent activity of GNC in vivo. Although a single qualified GNC batch was used throughout this study to minimize within‐study variability, formal inter‐batch chemical comparison was not performed. Future work should incorporate multi‐batch chromatographic fingerprinting and biological equivalence testing to further define the reproducibility of GNC preparations.

Additional limitations pertain to our pharmacological and mechanistic approaches. The use of GNC‐containing serum from ovariectomized rats, while consistent with established serum pharmacology methods, does not capture the dynamic hormonal fluctuations present in intact cycling females, which may influence both compound bioavailability and cellular responsiveness (Kharode et al. 2008). Furthermore, although molecular docking predictions were supported by the inhibitory effect of GNC‐containing serum on PTP1B enzymatic activity, the binding affinity and inhibitory potency of individual compounds, such as paeoniflorin, remain unquantified. Future studies employing biophysical techniques—such as surface plasmon resonance and kinetic inhibition assays—will be important to validate these interactions and define relative contributions.

Looking forward, the identification of ovarian PTP1B as a central regulatory node offers promising translational opportunities. It could serve as both a therapeutic target and a biomarker for women at risk of metabolically accelerated reproductive decline. Advanced approaches, such as chemical proteomics to map the direct protein targets of GNC in granulosa cells and single‐cell RNA sequencing to characterize heterogeneous ovarian responses, will further refine our understanding of PTP1B's role and inform the development of GNC‐based interventions for preserving reproductive longevity.

## 5 Conclusion

In summary, this study defines a PTP1B‐centered pathway linking metabolic stress to aging‐relevant ovarian dysfunction. Metabolic stress upregulates ovarian PTP1B, impairs granulosa‐cell insulin sensitivity, and contributes to follicular loss. GNC suppresses PTP1B, restores insulin signaling through the IRS1–AKT2–GLUT4 axis, and improves granulosa‐cell function, thereby preserving ovarian metabolic homeostasis under metabolic challenge. These findings identify PTP1B as a pivotal mediator connecting systemic metabolic stress with ovarian functional decline and support PTP1B‐targeted intervention as a potential strategy for protecting ovarian function in metabolically vulnerable states.

## Author Contributions

**Yanqiu Rao:** conceptualization, investigation, formal analysis, writing – original draft. **Ting Xu:** conceptualization, investigation, methodology, writing – original draft. **Yan Ding:** resources, data curation, validation. **Jun Li:** resources, supervision. **Lingyun Gao:** resources, supervision. **Yun Wang:** supervision, funding acquisition, project administration, writing – review and editing. **Wenjun Wang:** supervision, funding acquisition, project administration, writing – review and editing. **Yanqiu Rao** and **Ting Xu** contributed equally to this work. All authors reviewed and approved the final version of the manuscript.

## Funding

This work was supported by the Science and Technology Commission of Shanghai Municipality under the Shanghai Science and Technology Innovation Action Plan (Grant No. 23Y11920300).

## Ethics Statement

All animal experiments were approved by the Institutional Animal Care and Use Committee of Fudan University (Approval No. 2024‐FCKYY‐084) and were performed in accordance with institutional guidelines. The human granulosa‐cell transcriptomic dataset analyzed in this study was publicly available from the NCBI Gene Expression Omnibus under accession number GSE232306. No new human participants were recruited for this study.

## Consent

The authors have nothing to report.

## Conflicts of Interest

The authors declare no conflicts of interest.

## Supporting information


**Data S1:** Author checklist.

**Table S1:** HPLC quantification of representative GNC marker compounds.
**Table S2:** Pharmacokinetic parameters of paeoniflorin after repeated GNC administration.
**Table S3:** Basic characteristics of the therapeutic intervention cohort.

**Figure S1:** HPLC‐based quality control of GNC granules.

**Figure S2:** Determination of the working concentration of GNC‐containing serum.

**Figure S3:** Validation of HFD‐induced systemic insulin resistance and metabolic stress‐associated ovarian dysfunction.

**Figure S4:** Optimization of insulin concentration for KGN‐IR model establishment.

**Figure S5:** GNC‐containing serum does not alter basal insulin signaling in insulin‐sensitive KGN cells.

Supporting Information S1.

## Data Availability

The transcriptomic dataset analyzed in this study (GSE232306) is publicly available in the NCBI GEO repository. The PTP1B crystal structure analyzed in this study (PDB: 7MNF) is available from the Protein Data Bank. All additional data supporting the findings of this study, including uncropped Western blot images with molecular weight markers and raw densitometric quantification data, are provided as Supporting Information Raw Data files accompanying the revised submission.
