# Supplementary material for: Protein Tyrosine Phosphatase 1B‐Mediated Granulosa Cell Insulin Resistance Links Metabolic Stress to Aging‐Relevant Ovarian Dysfunction and Is Reversed by Gengnianchun
Source: Aging Cell. 2026 Jun 9;25(6):e70583. doi: 10.1111/acel.70583 (PMC13249799; doi:10.1111/acel.70583)
Supplement: Supplementary file 1 — Data S1: Author checklist. [file ACEL-25-e70583-s001.docx]

**Author Checklist**

**Title**

Protein tyrosine phosphatase 1B-mediated granulosa cell insulin resistance links metabolic stress to aging-relevant ovarian dysfunction and is reversed by Gengnianchun

**Authors**Yanqiu Rao^a,b,1^, Ting Xu^a,b,1^, Yan Ding^c^, Jun Li^a,b^, Lingyun Gao^a,b^, Yun Wang^a,b,*^, Wenjun Wang^a,b,*^

**Total word count**

7044

**Word count of Summary**223

**Number of papers cited in the References**42

**Listing of all Tables**Table 1. Composition of Gengnianchun formula

**Listing of all Figures**

| **Checklist** | **Figure Preparation Guidelines and Tips: Preferred standards for peer review and required standards for production.** |
| --- | --- |
| √ | Are all figures included in your submission as separate files or in a single PDF/Word document/LaTeX suite? **Tip!** Single, original, unconverted files are best. |
| √ | Do all figures have an accompanying legend that describes the content and explains any abbreviations or symbols? **Tip!** Include your figure legends as a separate section in your main text file. |
| √ | Are all figures cited in the main text of your article? **Tip!** Ensure all figures are numbered in the order in which they appear. |
| √ | Are all words or symbols in your figures large enough for easy reading by your audience? **Tip!** Closely follow the preferred resolution guidelines for best presentation. |
| √ | Are all figures saved in an acceptable file type? **Tip!** Use the preferred file types for best image quality. If in doubt, submit a PDF for initial review. |
| √ | Is each individual figure file less than 10 MB? **Tip!** Remove excess white space surrounding figures for smaller file sizes. |
| √ | Were figures created between 80 and 180 mm width and at 300 to 600 DPI? **Tip!** Higher quality figures are more useful to readers. |
| √ | Are all figure files named with their appropriate figure number? **Tip!** Using only figure numbers in the file names ensures correct typesetting. |

**Supporting Information:**

Supplementary Figures S1–S5;

Supplementary Tables S1–S3;

Supplementary Raw Data containing uncropped Western blot images.
