# Supplementary material for: Protein Tyrosine Phosphatase 1B‐Mediated Granulosa Cell Insulin Resistance Links Metabolic Stress to Aging‐Relevant Ovarian Dysfunction and Is Reversed by Gengnianchun
Source: Aging Cell. 2026 Jun 9;25(6):e70583. doi: 10.1111/acel.70583 (PMC13249799; doi:10.1111/acel.70583)
Supplement: Supplementary file 2 — Table S1: HPLC quantification of representative GNC marker compounds. Table S2: Pharmacokinetic parameters of paeoniflorin after repeated GNC administration. Table S3: Basic characteristics of the therapeutic intervention cohort. [file ACEL-25-e70583-s005.docx]

**Supplementary Figure S1 | HPLC-based quality control of GNC granules.**Representative HPLC-DAD chromatograms of marker compounds in GNC granules from batch no. 1910304. (A) Paeoniflorin standard, methanol extract, and diluted ethanol extract. (B) Berberine standard, methanol extract, and diluted ethanol extract. (C) Icariin standard, methanol extract, and diluted ethanol extract. Detection wavelengths were 230 nm for paeoniflorin, 345 nm for berberine, and 270 nm for icariin. Marker-compound contents are provided in Supplementary Table S1.

**Supplementary Figure S2 | Determination of the working concentration of GNC-containing serum.**KGN cells were treated with increasing concentrations of GNC-containing serum for 48 h, and cell viability was assessed using the CCK-8 assay. Viability was normalized to untreated control cells and plotted on a log2 concentration scale. GNC-containing serum at 10% (v/v) was selected for subsequent in vitro experiments.

**Supplementary Figure S3 | Validation of HFD-induced systemic insulin resistance and metabolic stress-associated ovarian dysfunction.**
Female C57BL/6J mice were fed a standard chow diet or a 45% kcal HFD for 6 weeks before therapeutic intervention. (A) Body-weight changes during the modelling period and final body weight. (B) IPGTT and ITT curves with corresponding AUC analyses. (C) Fasting insulin levels and HOMA-IR. (D) Serum FSH, LH, E2, and AMH. (E) Estrous-cycle stage distribution and estrus frequency. (F) Quantification of ovarian follicles and corpora lutea. Data are mean ± SD; n = 8 mice per group. *p < 0.05, **p < 0.01 versus Control.

**Supplementary Figure S4 | Optimization of insulin concentration for KGN-IR model establishment.**

KGN cells were exposed to recombinant human insulin at 10–640 nM for 48 h, and glucose consumption was measured to determine the induction condition for the KGN-IR model. Insulin at 160 nM for 48 h was selected for subsequent experiments. Data are mean ± SD from at least three independent experiments. *p < 0.05, **p < 0.01 versus Control.

**Supplementary Figure S5 | GNC-containing serum does not alter basal insulin signaling in insulin-sensitive KGN cells.**

Insulin-sensitive KGN cells were treated with 10% GNC-containing serum or insulin for 6 h. (A) Glucose consumption after treatment with GNC-containing serum or insulin at the indicated concentrations. (B) IF staining and quantification of GLUT4 fluorescence. GLUT4, red; DAPI, blue. (C) Western blot analysis of GLUT4 and IRS1/AKT2/mTOR signalling. Phosphorylated IRS1, AKT2, and mTOR were quantified as p/total protein ratios and as p/β-actin ratios; GLUT4 was normalized to β-actin. Scale bars, 50 μm. Data are mean ± SD from at least three independent experiments. *p < 0.05, **p < 0.01 versus Control.

**Supplementary Table S1 | HPLC quantification of representative GNC marker compounds.**

| Marker compound | Calibration equation | R² | Peak area (MeOH) | Peak area (dilute EtOH) | Concentration (MeOH, μg/mL) | Concentration (dilute EtOH, μg/mL) | Content (MeOH, %) | Content (dilute EtOH, %) |
| --- | --- | --- | --- | --- | --- | --- | --- | --- |
| Paeoniflorin | y=0.292x+0.08 | 0.9997 | 10.2744 | 12.9831 | 34.91 | 44.19 | 0.35 | 0.44 |
| Berberine | y=0.8397x+0.2632 | 0.9995 | 25.4617 | 32.7513 | 30.64 | 39.32 | 0.31 | 0.39 |
| Icariin | y=0.581x+0.069 | 1 | 5.1223 | 6.2087 | 8.70 | 10.57 | 0.09 | 0.11 |

Paeoniflorin, berberine, and icariin were quantified by HPLC-DAD in GNC batch no. 1910304. Methanol and dilute ethanol extracts were analyzed in parallel. The table shows calibration equations, R² values, peak areas, calculated concentrations, and compound contents. GNC, Gengnianchun formula; MeOH, methanol; EtOH, ethanol.

**Supplementary Table S2 | Pharmacokinetic parameters of paeoniflorin after repeated GNC administration.**

| Parameter | Value (mean ± SD) |
| --- | --- |
| T1/2 (h) | 7.16±4.56 |
| Tmax (h) | 1.71±0.95 |
| Cmax (ng/mL) | 41.20±14.54 |
| AUC(0-t) (h*(ng/mL)) | 272.13±108.75 |
| MRT(0-t) (h) | 6.32±1.86 |

The table summarizes T1/2, Tmax, Cmax, AUC(0–t), and MRT(0–t) of paeoniflorin in Sprague–Dawley rats after repeated oral administration of GNC granules. Data are presented as mean ± SD. GNC, Gengnianchun formula.

**Supplementary Table S3 | Basic characteristics of the therapeutic intervention cohort.**

| Characteristic | Control | HFD | HFD + GNC | HFD + MET |
| --- | --- | --- | --- | --- |
| Strain / source | C57BL/6J / Shanghai SLAC | C57BL/6J / Shanghai SLAC | C57BL/6J / Shanghai SLAC | C57BL/6J / Shanghai SLAC |
| n | 8 | 8 | 8 | 8 |
| Sex | Female | Female | Female | Female |
| Age (weeks) | 6-8 | 6-8 | 6-8 | 6-8 |
| Diet | Standard chow | HFD 45% kcal fat | HFD 45% kcal fat | HFD 45% kcal fat |
| Modeling period | — | 6 weeks HFD | 6 weeks HFD | 6 weeks HFD |
| Route of administration | Oral gavage | Oral gavage | Oral gavage | Oral gavage |
| Intervention / Treatment | Vehicle 6 weeks | Vehicle 6 weeks | GNC 1.4 g/kg/day 6 weeks | Metformin 200 mg/kg/day 6 weeks |
| Randomization | Yes | Yes | Yes | Yes |
| Blinding | Yes | Yes | Yes | Yes |
| Initial body weight (g) | 14.95 ± 0.25 | 14.88 ± 0.25 | 15.00 ± 0.33 | 14.85 ± 0.27 |
| Mortality / Exclusion | 0 | 0 | 0 | 0 |

The table summarizes animal strain, source, group size, sex, age, diet, modeling period, administration route, intervention, randomization, blinding, initial body weight, and mortality or exclusion information for Control, HFD, HFD + GNC, and HFD + metformin groups. HFD, high-fat diet; MET, metformin; GNC, Gengnianchun formula.
