# Supplementary figures and images for: Protein Tyrosine Phosphatase 1B‐Mediated Granulosa Cell Insulin Resistance Links Metabolic Stress to Aging‐Relevant Ovarian Dysfunction and Is Reversed by Gengnianchun
Source: Aging Cell. 2026 Jun 9;25(6):e70583. doi: 10.1111/acel.70583 (PMC13249799; doi:10.1111/acel.70583)

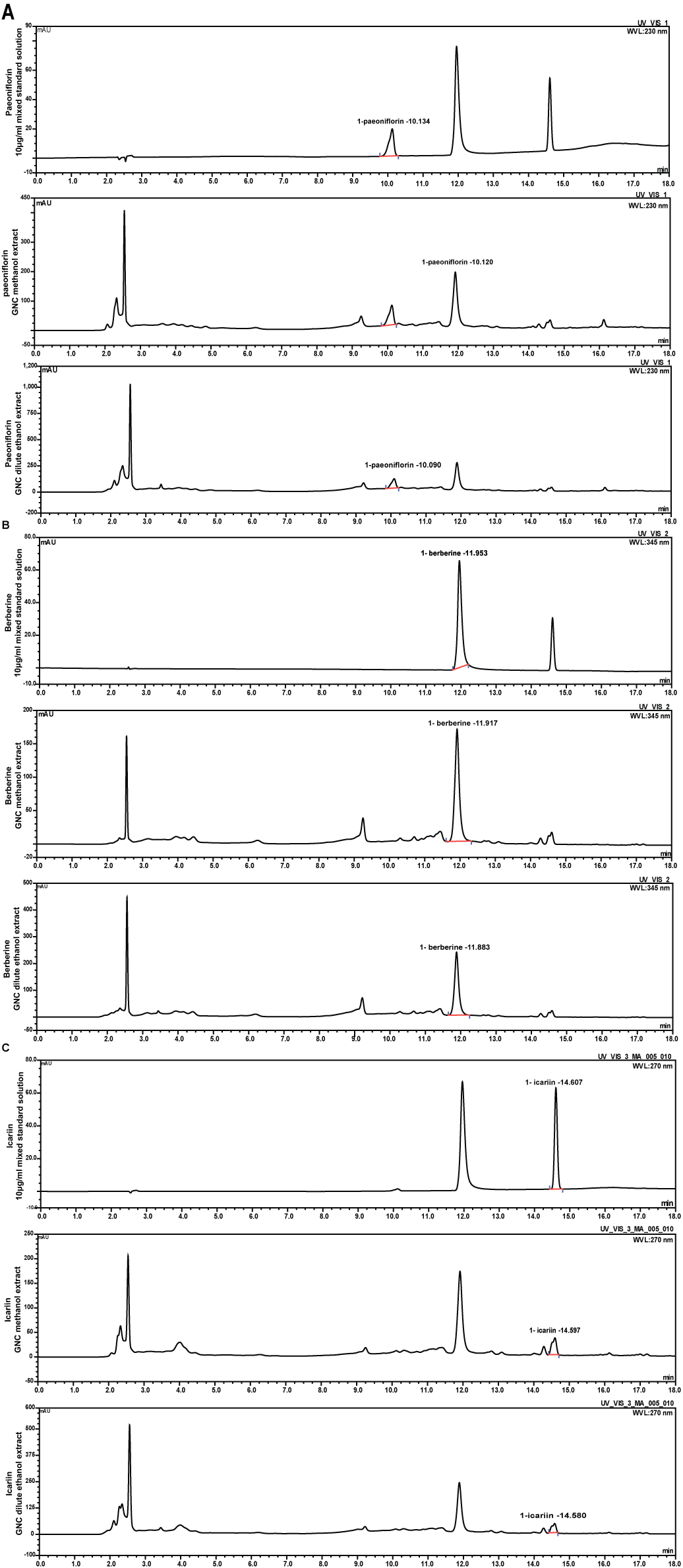

Supplement: Supplementary file 3 — Figure S1: HPLC‐based quality control of GNC granules. [file ACEL-25-e70583-s003.tif]

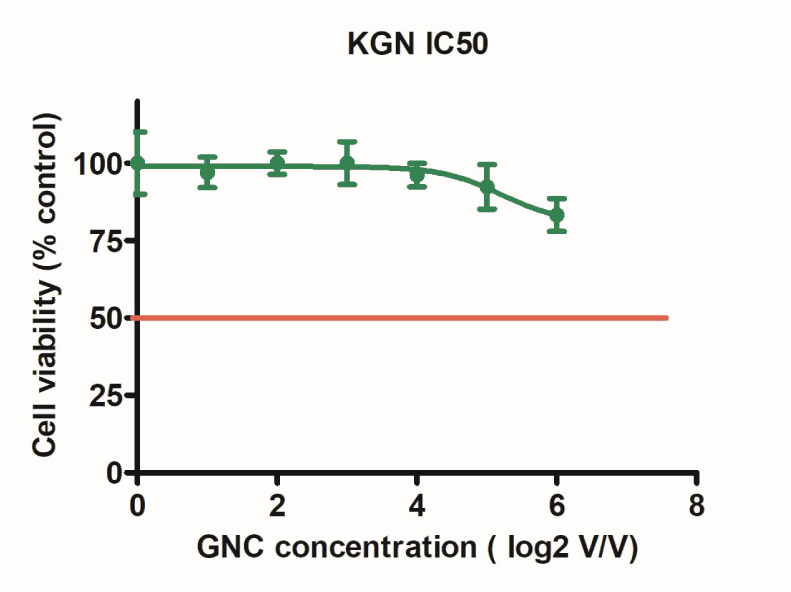

Supplement: Supplementary file 4 — Figure S2: Determination of the working concentration of GNC‐containing serum. [file ACEL-25-e70583-s002.tif]

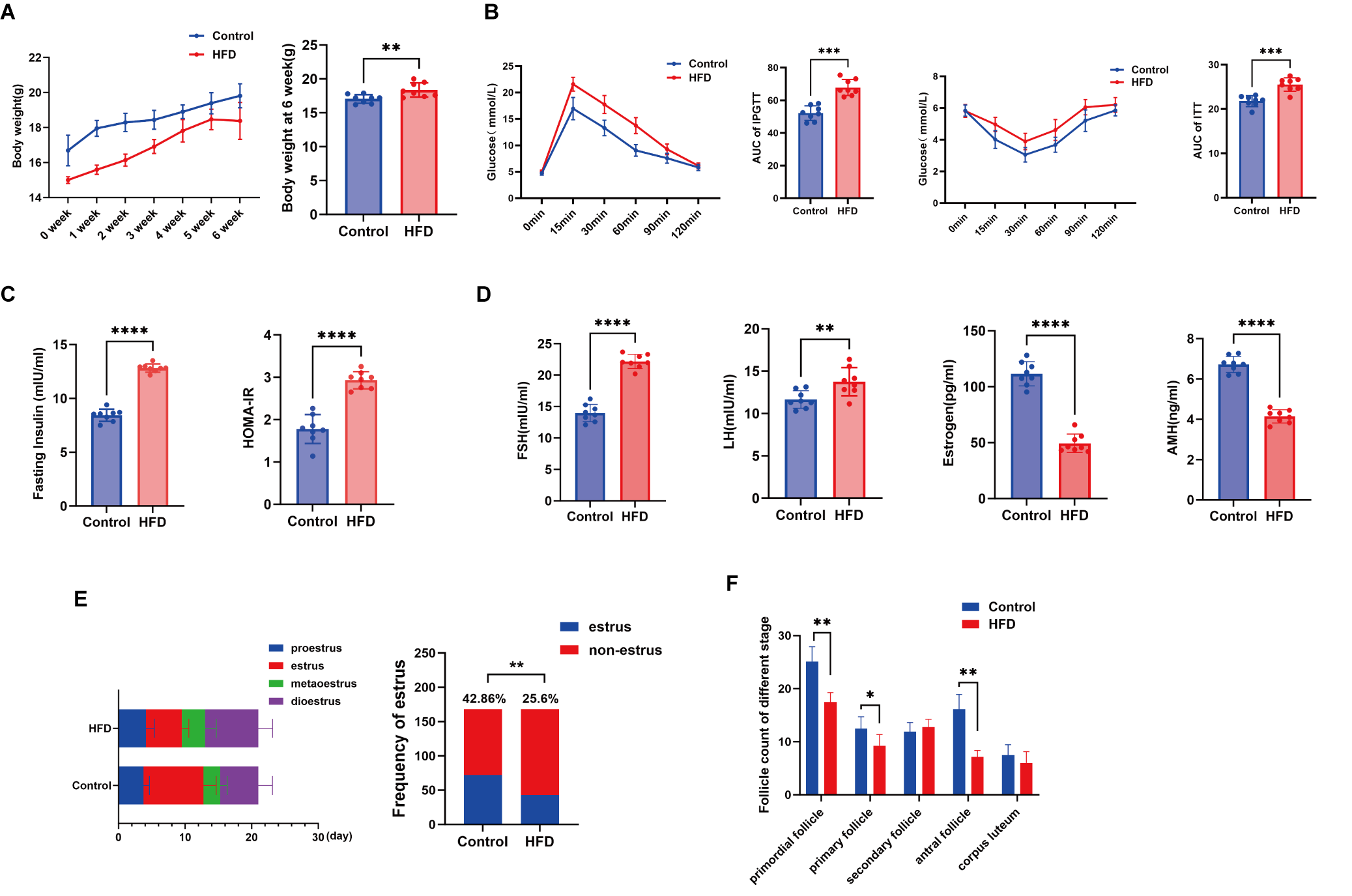

Supplement: Supplementary file 5 — Figure S3: Validation of HFD‐induced systemic insulin resistance and metabolic stress‐associated ovarian dysfunction. [file ACEL-25-e70583-s006.tif]

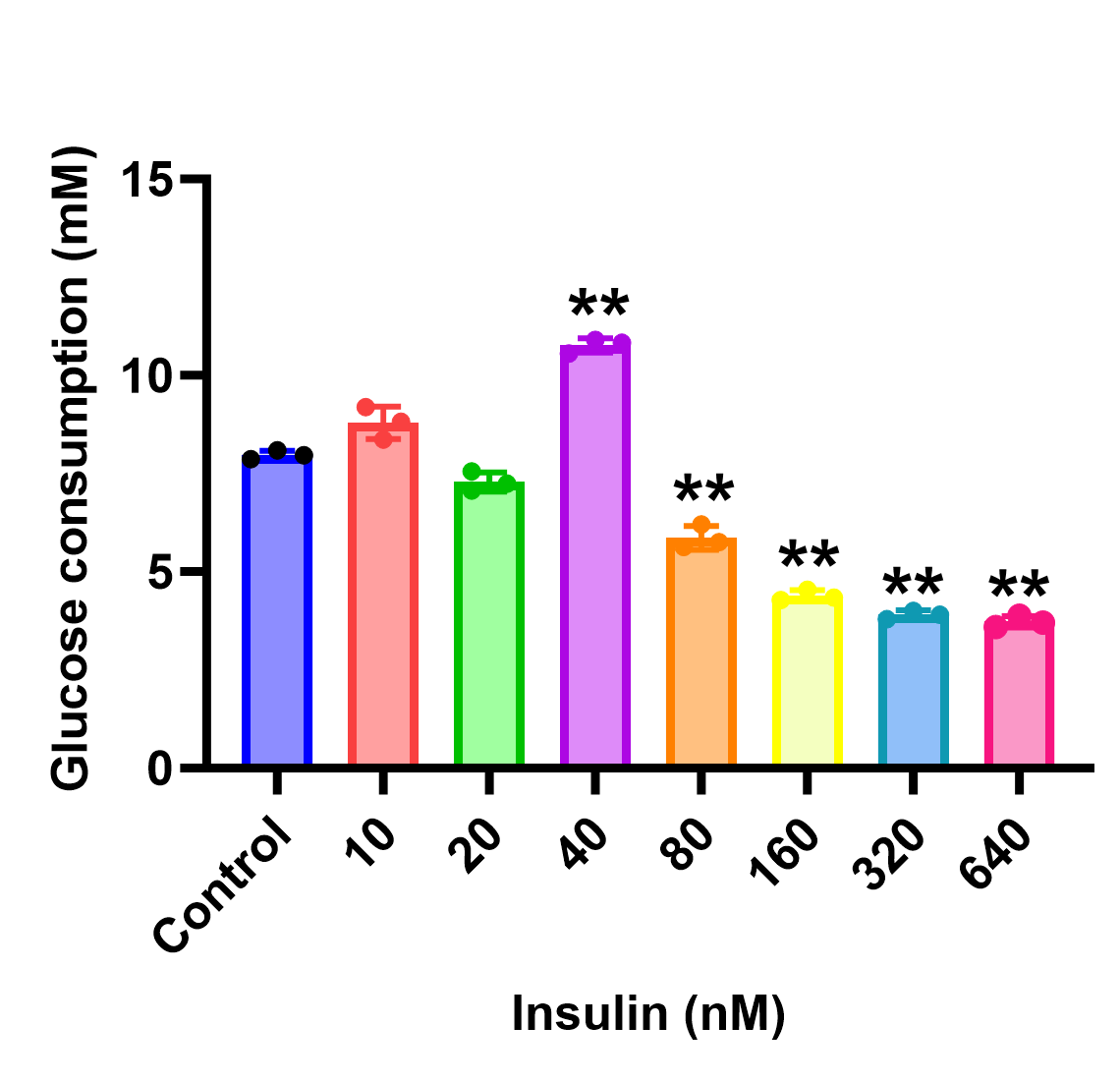

Supplement: Supplementary file 6 — Figure S4: Optimization of insulin concentration for KGN‐IR model establishment. [file ACEL-25-e70583-s007.tif]

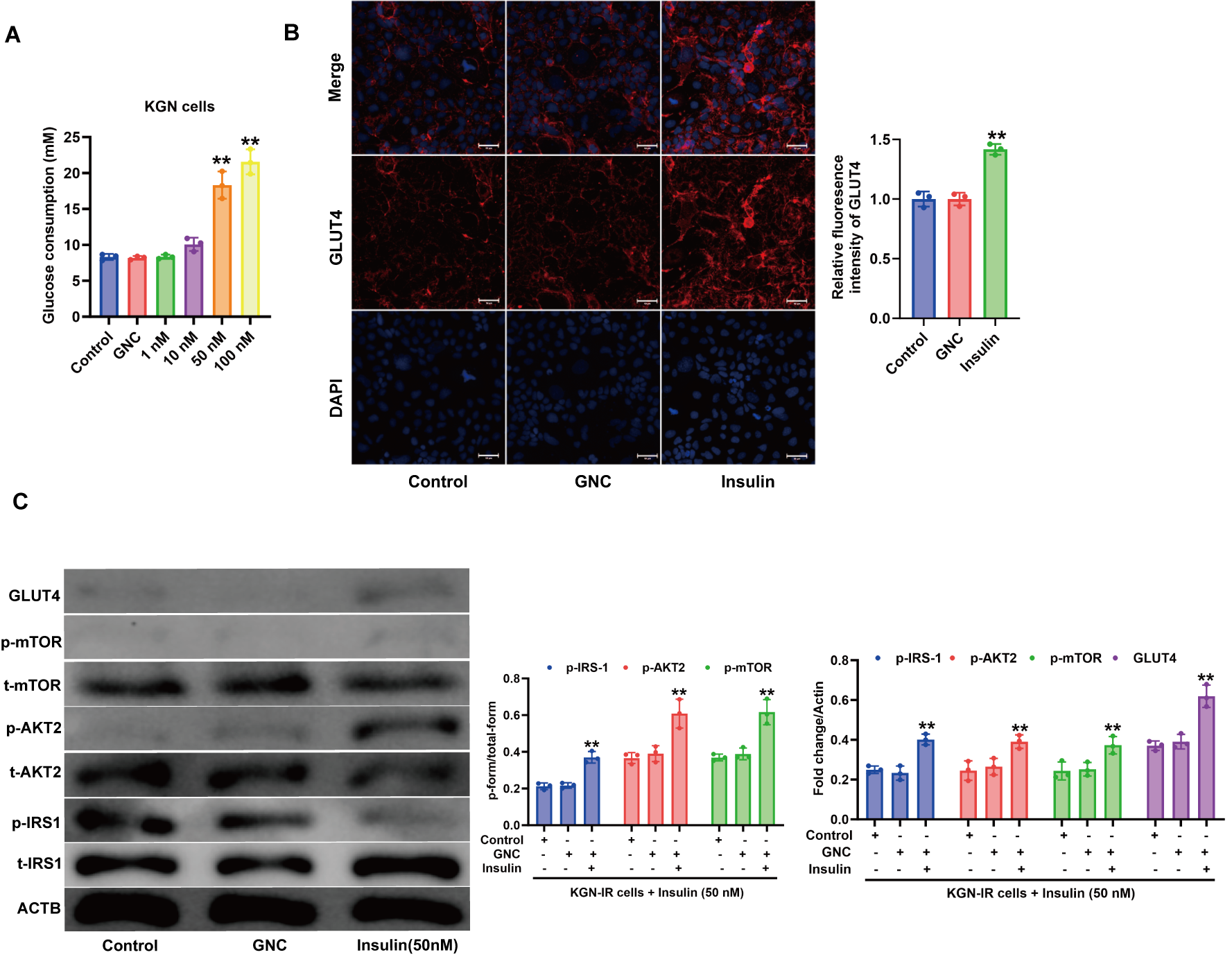

Supplement: Supplementary file 7 — Figure S5: GNC‐containing serum does not alter basal insulin signaling in insulin‐sensitive KGN cells. [file ACEL-25-e70583-s004.tif]
