# Supplementary material for: Protein Tyrosine Phosphatase 1B‐Mediated Granulosa Cell Insulin Resistance Links Metabolic Stress to Aging‐Relevant Ovarian Dysfunction and Is Reversed by Gengnianchun
Source: Aging Cell. 2026 Jun 9;25(6):e70583. doi: 10.1111/acel.70583 (PMC13249799; doi:10.1111/acel.70583)
Supplement: Supplementary file 8 — Supporting Information S1. [file ACEL-25-e70583-s008.docx]

Figure 3B

β-actin


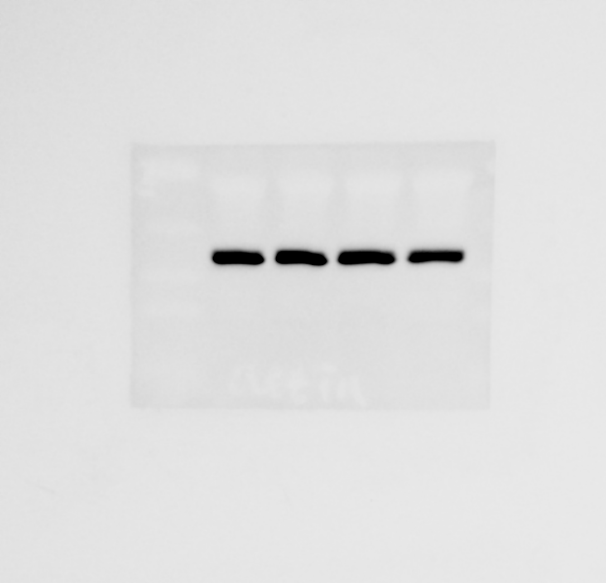

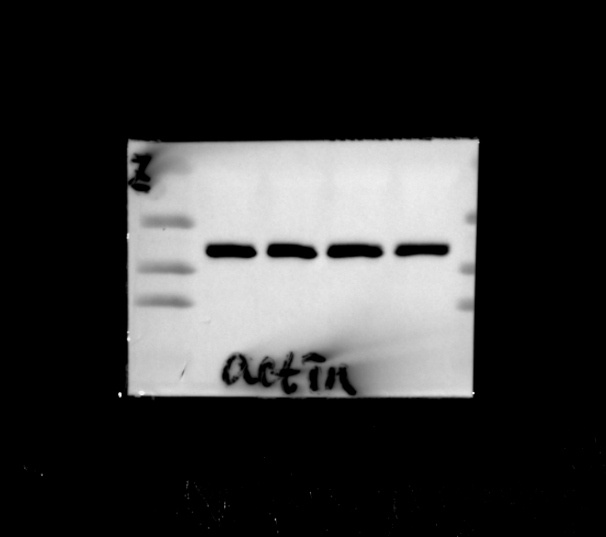


PTP1B


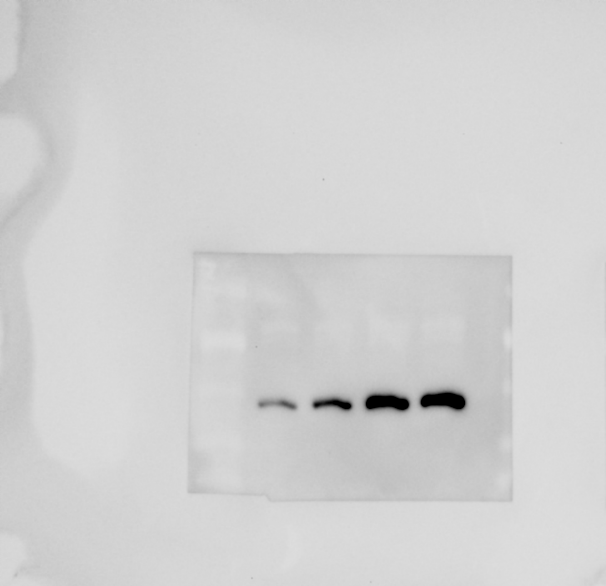

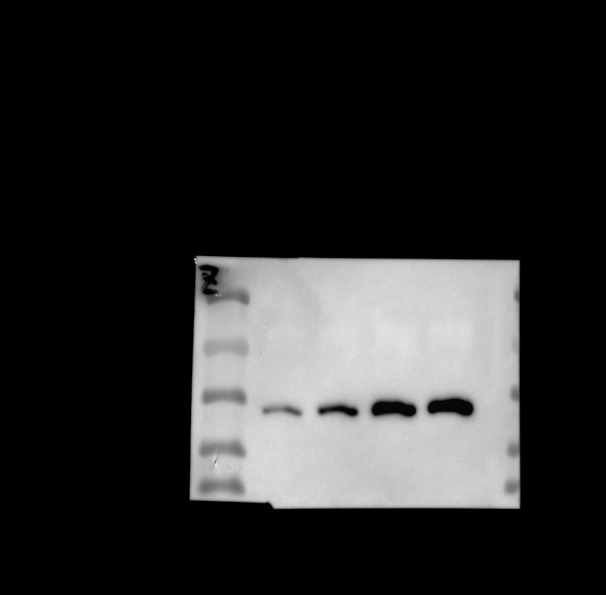


Figure 3C

β-actin


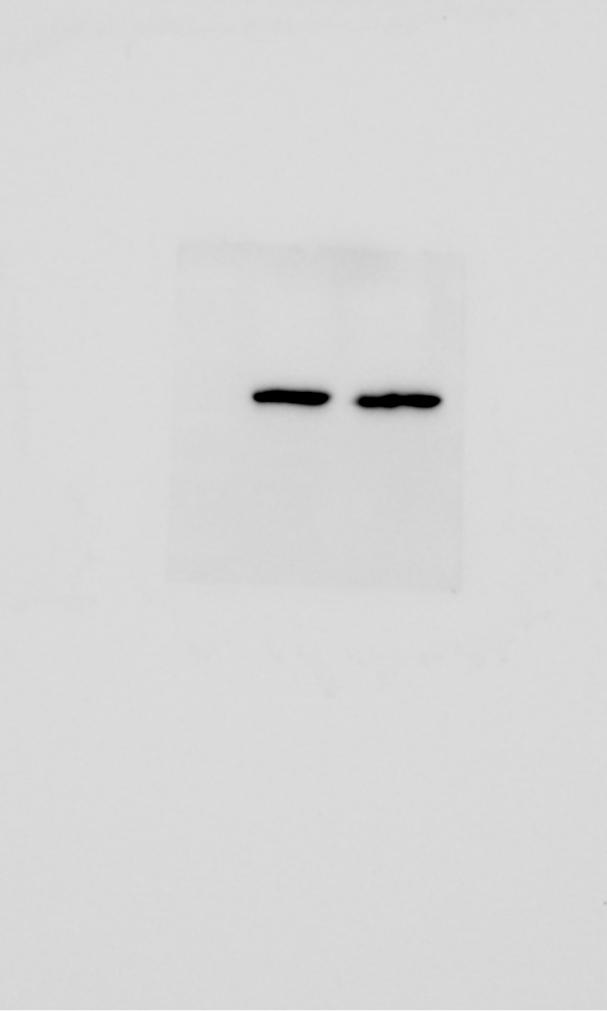

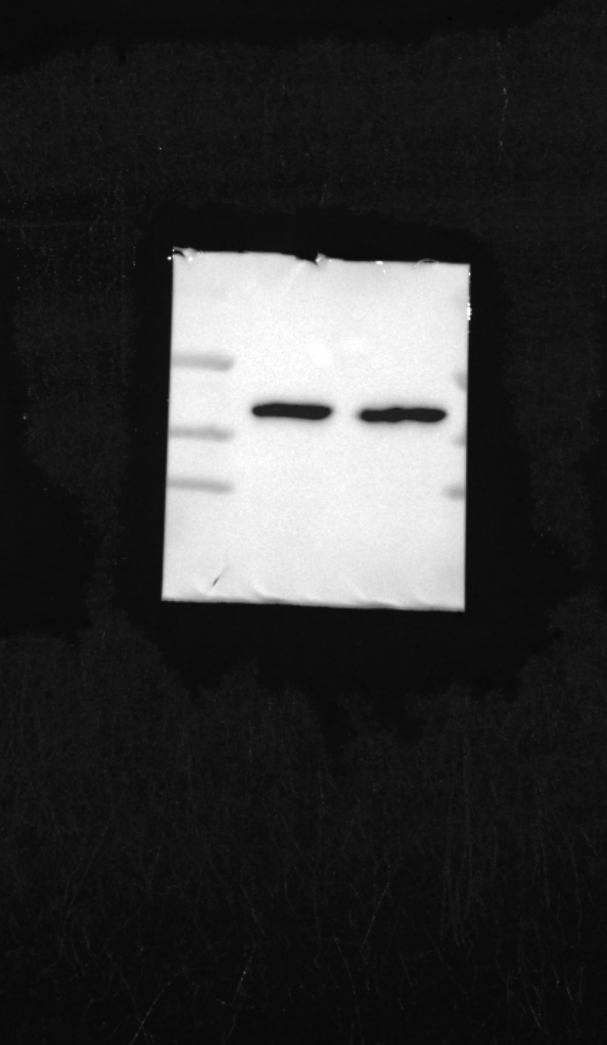


IRS1


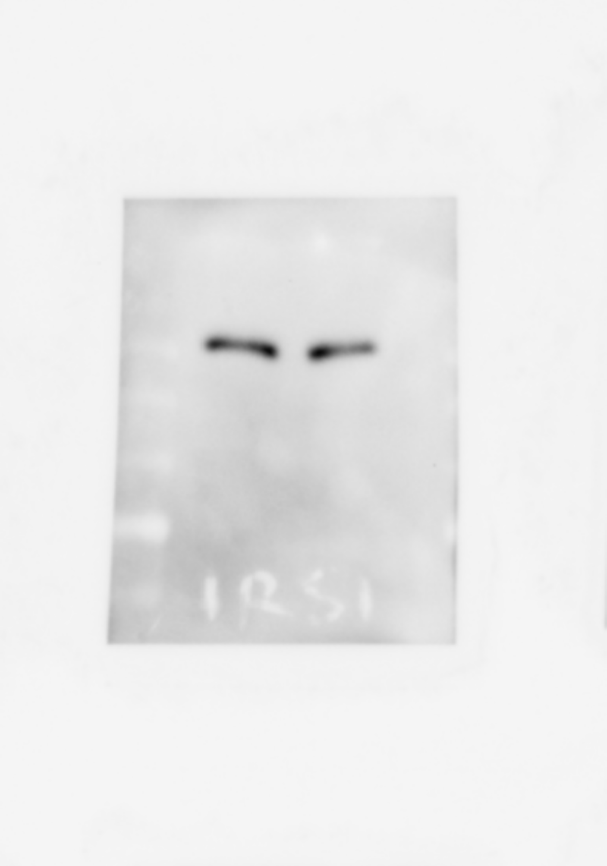

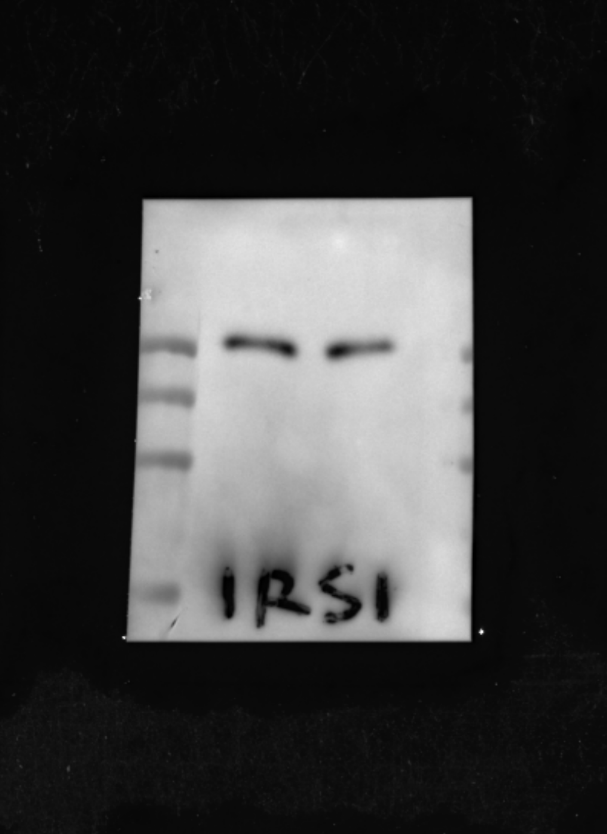


p-IRS1


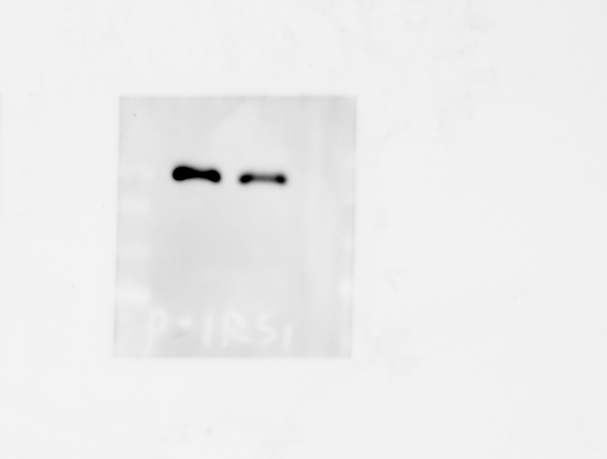

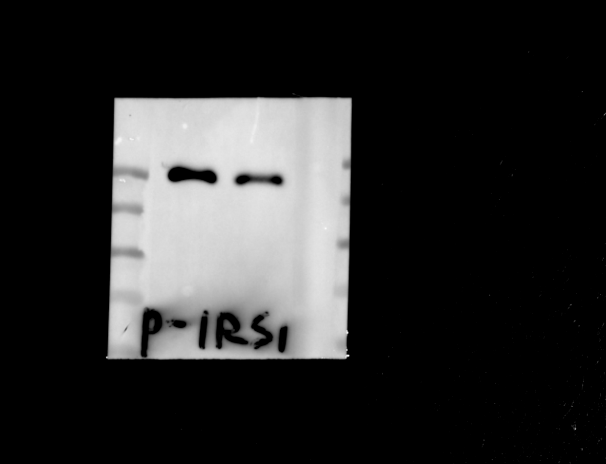


p-AKT2


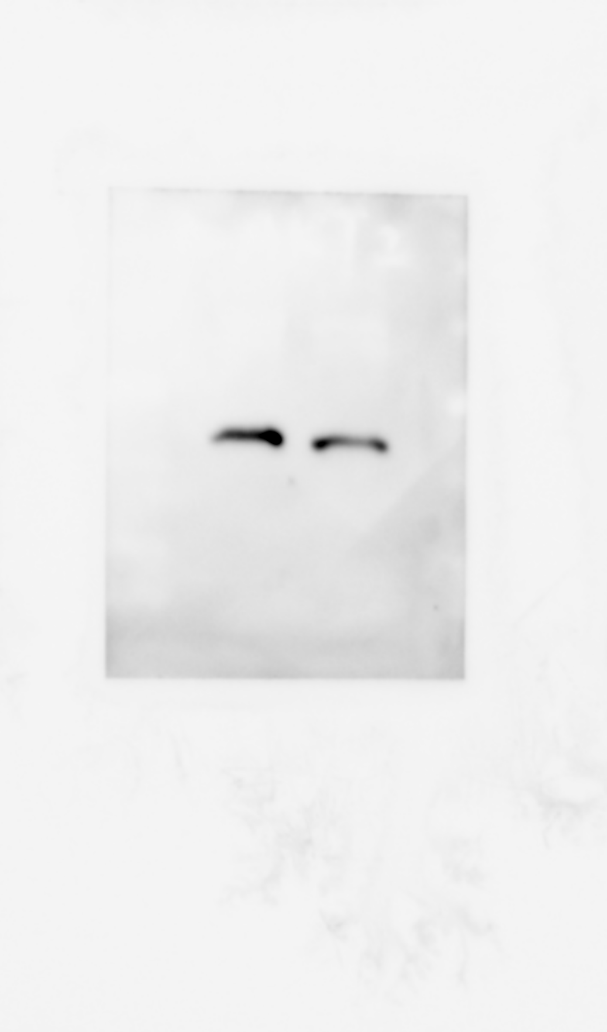

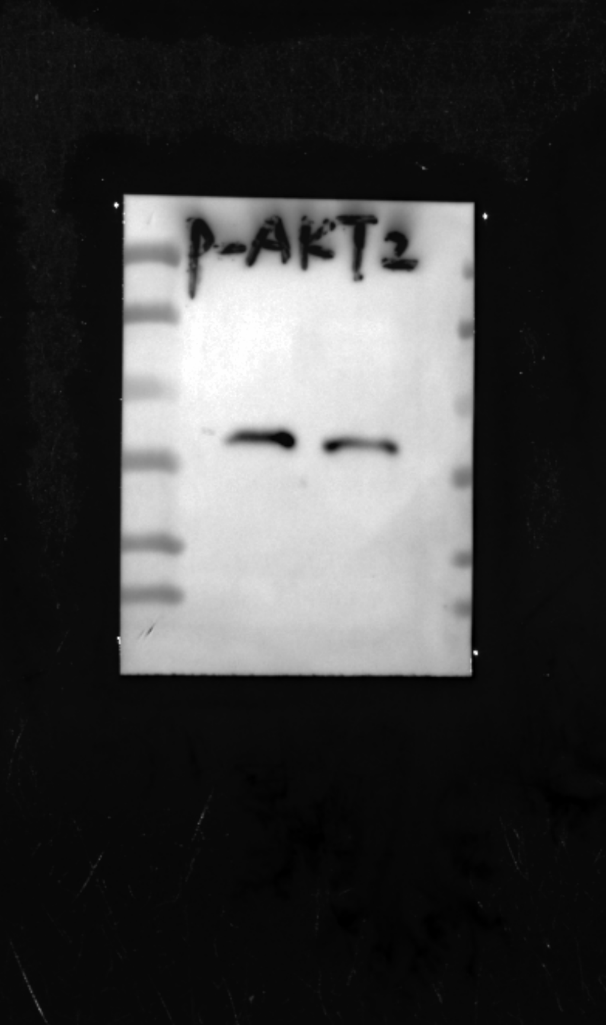


t-AKT2


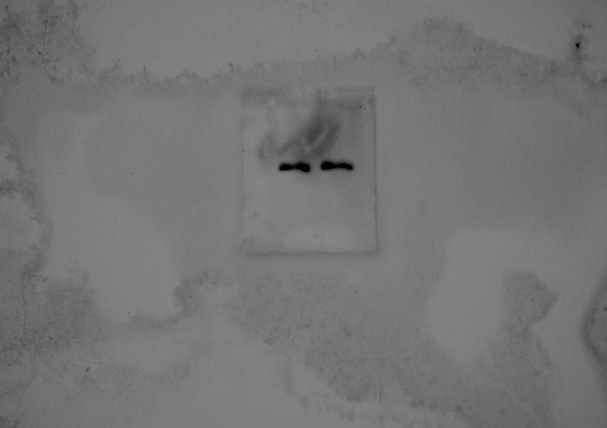

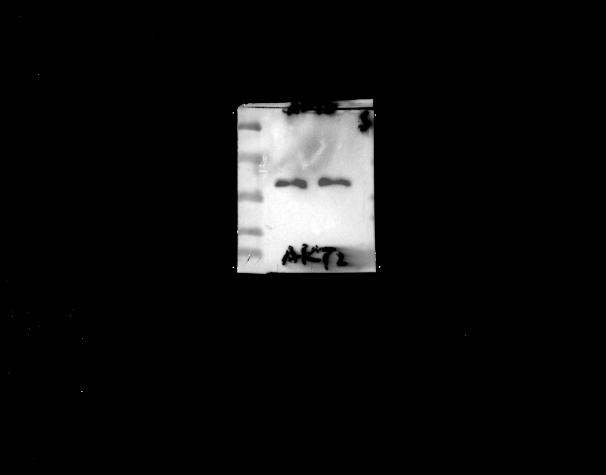


p-mTOR


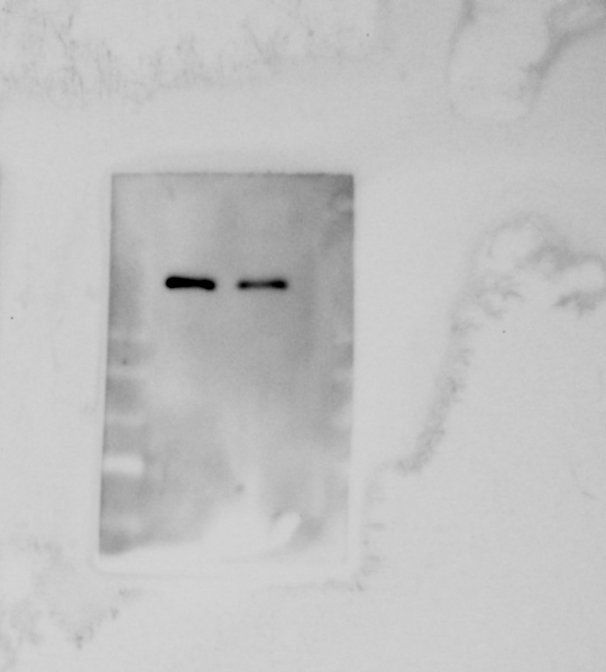

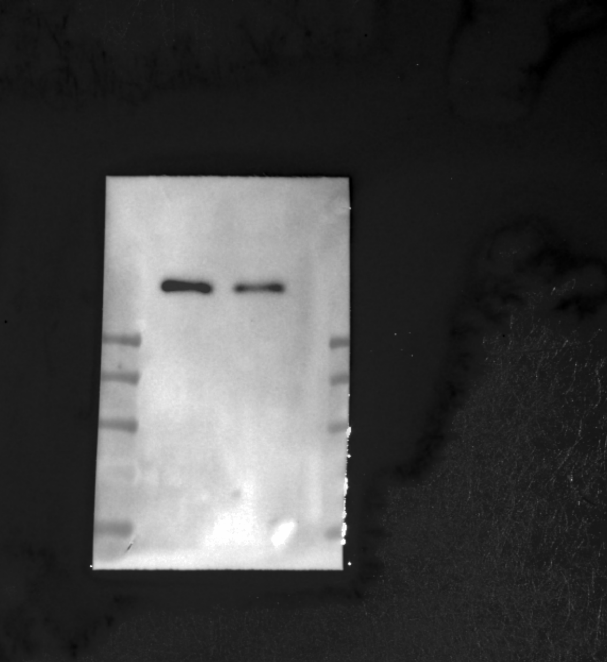


t-mTOR


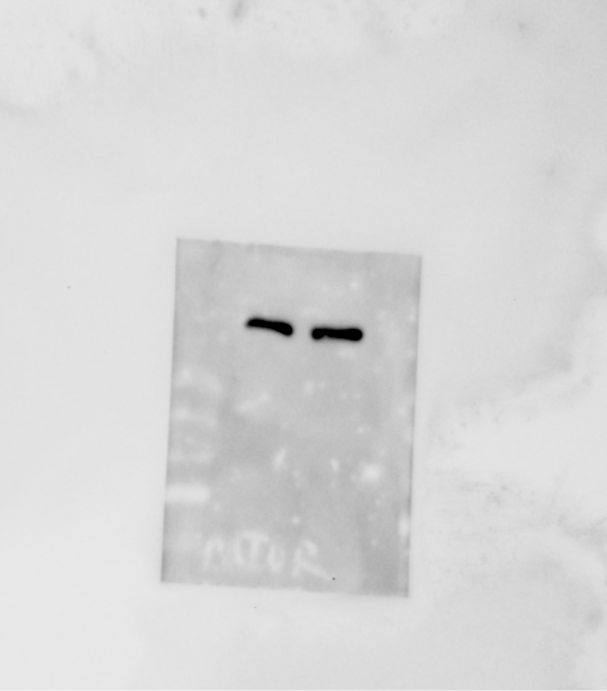

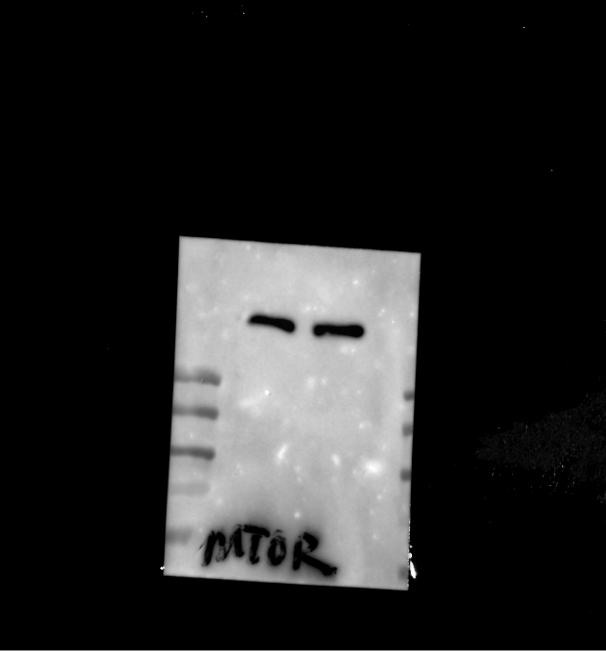


GLUT4


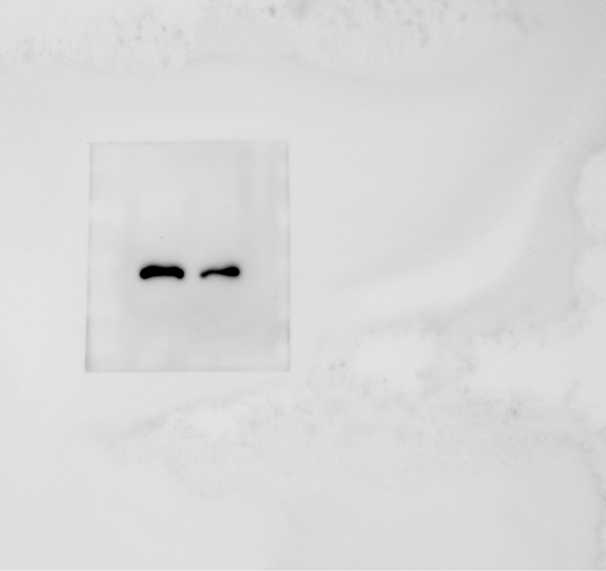

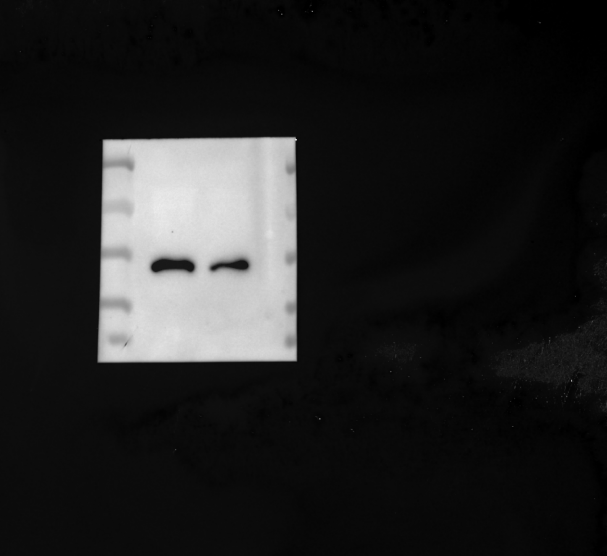


Figure 4B

β-actin


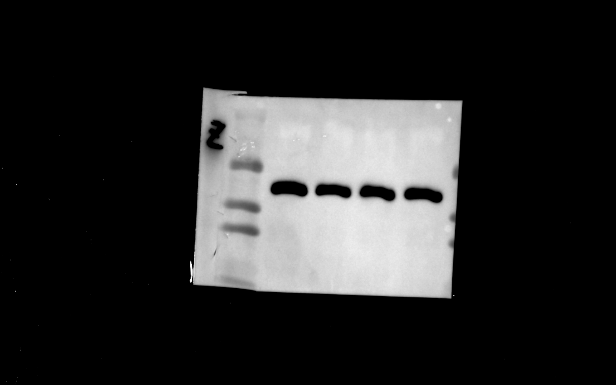

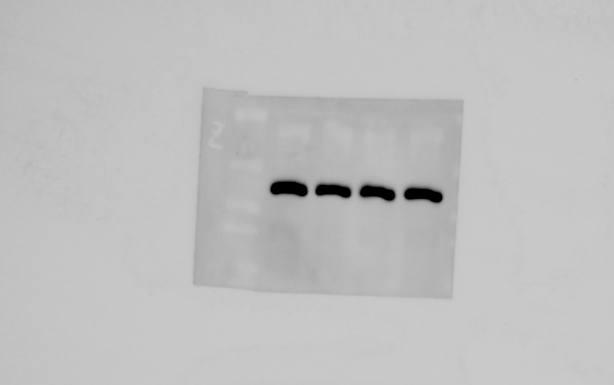


p‑IRS1


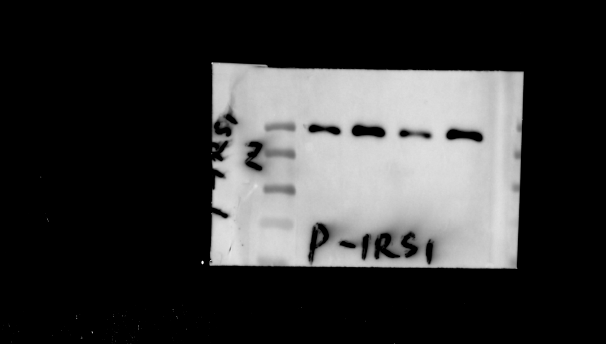

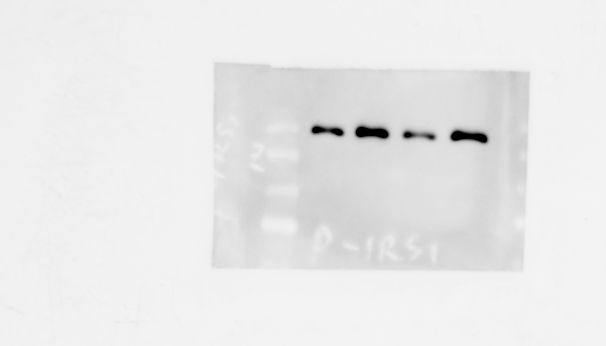


t‑IRS1


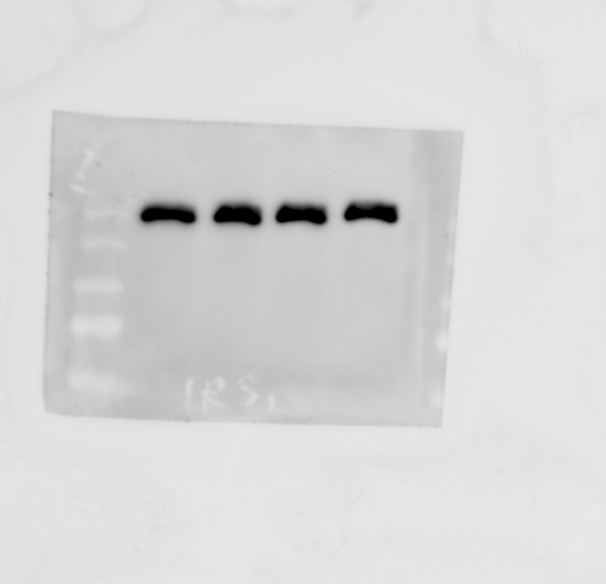

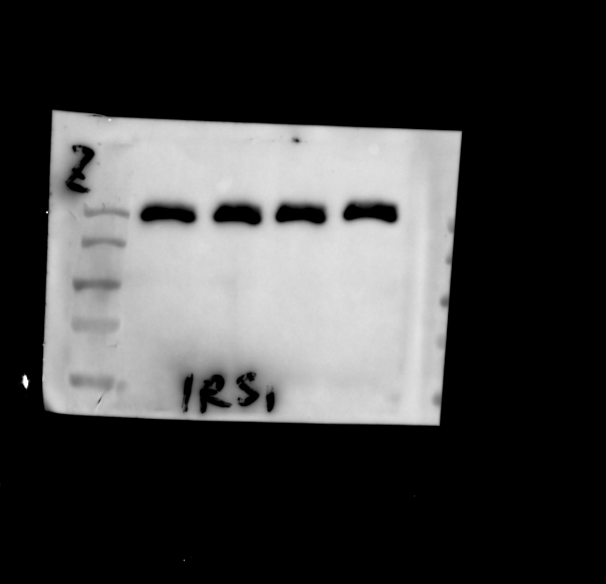


p‑AKT2


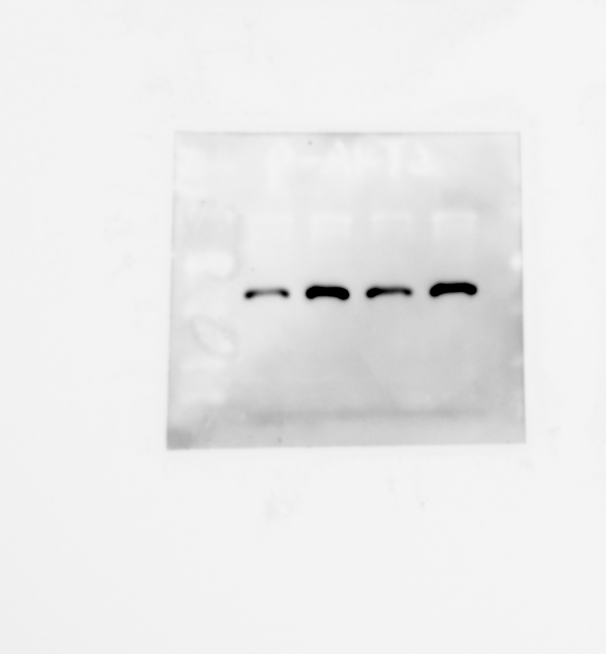

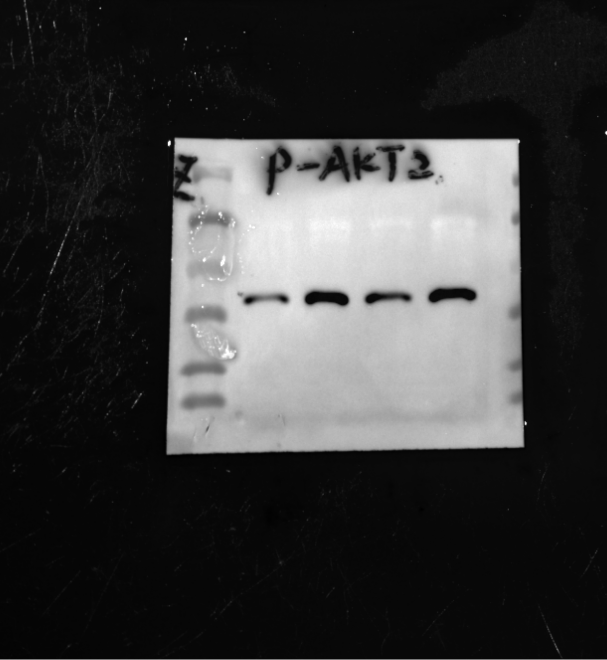


t‑AKT2


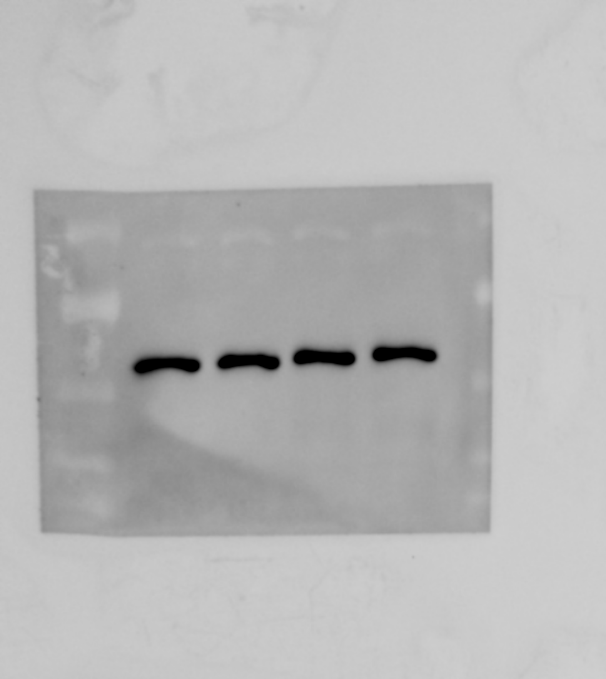

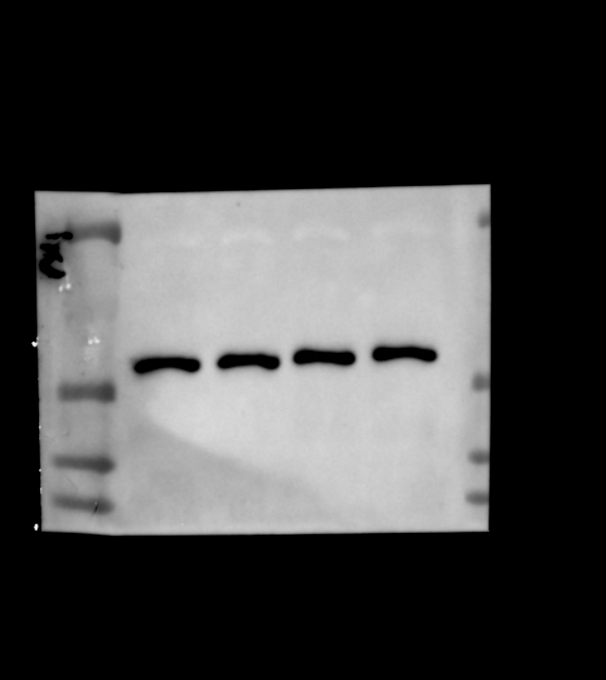


p‑mTOR


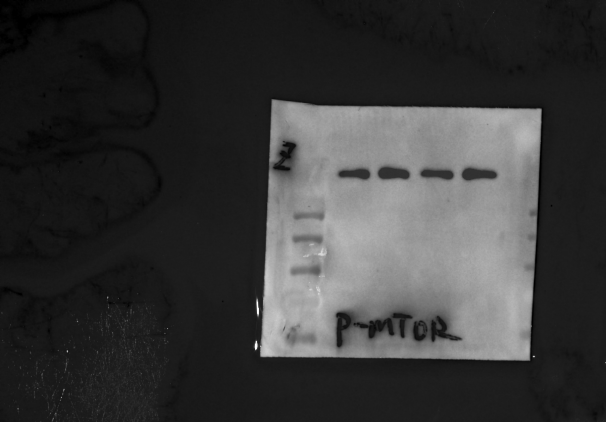

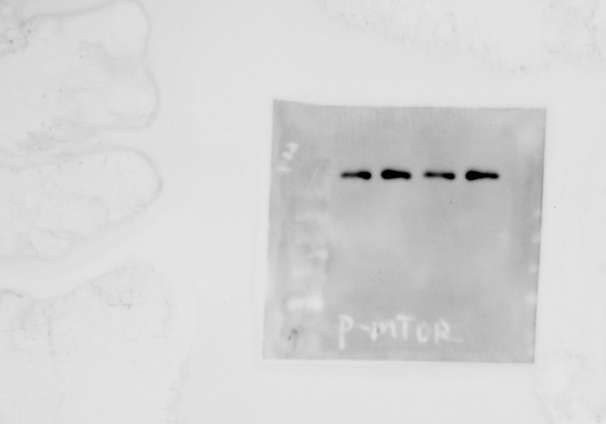


t‑mTOR


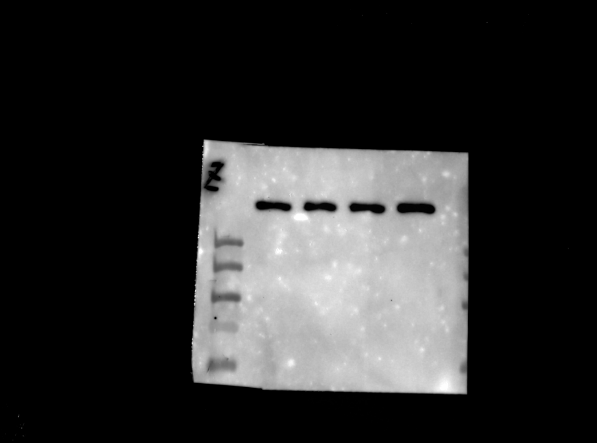

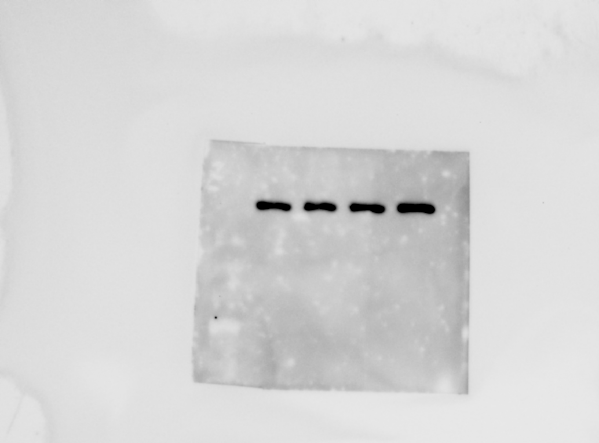


GLUT4


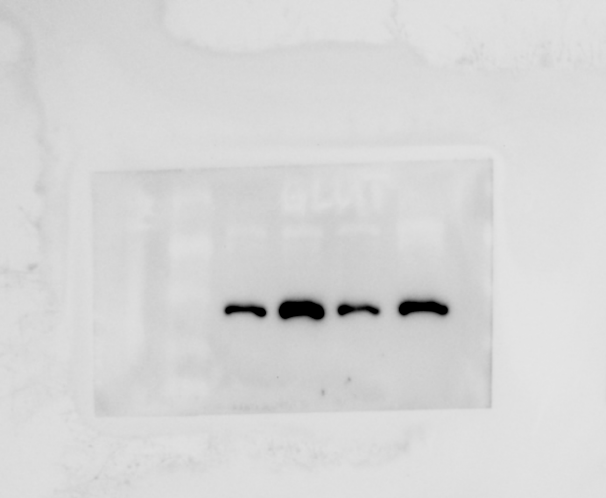

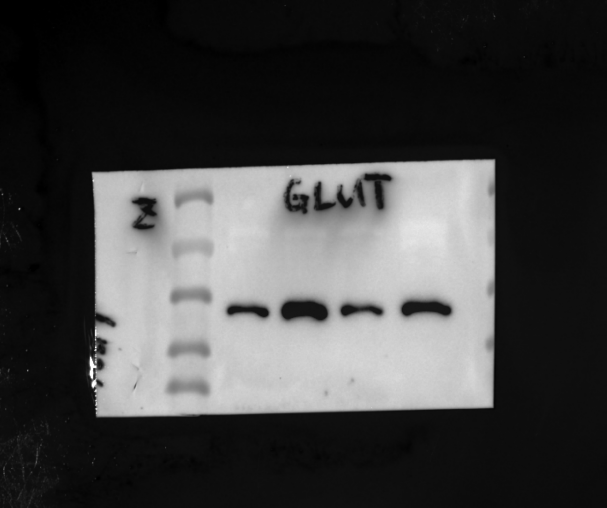


Figure 5B

β-actin


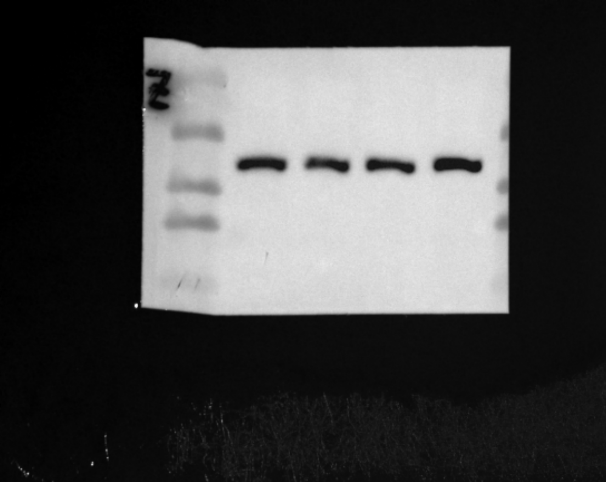

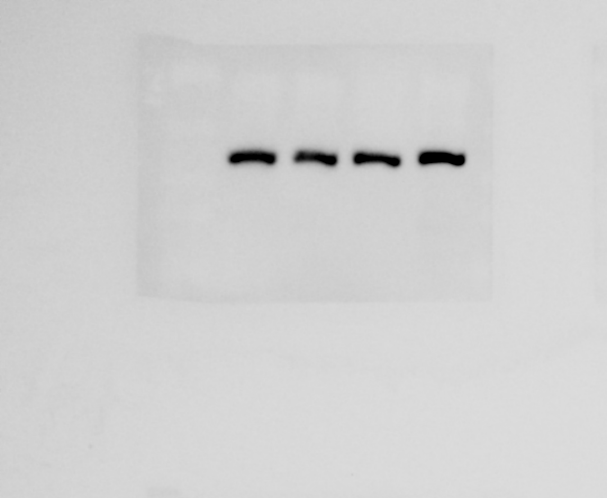


PTP1B


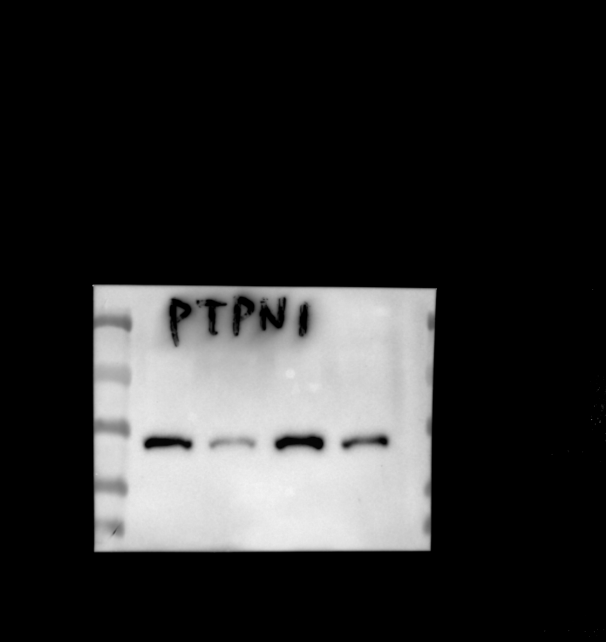

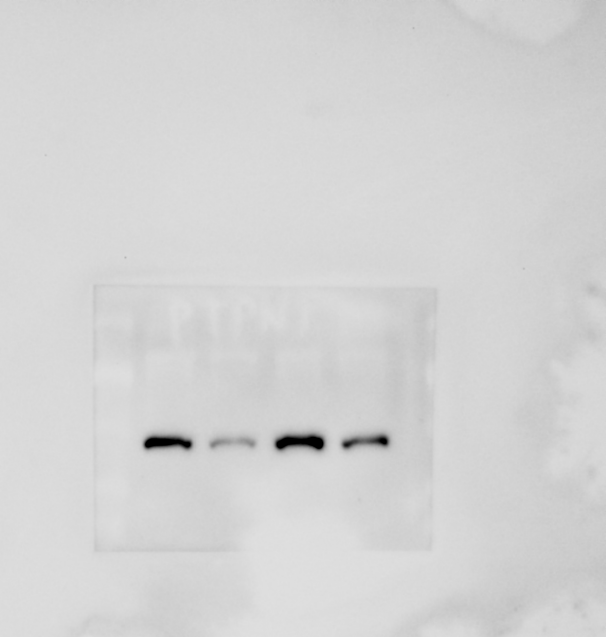


Figure 5D

β-actin


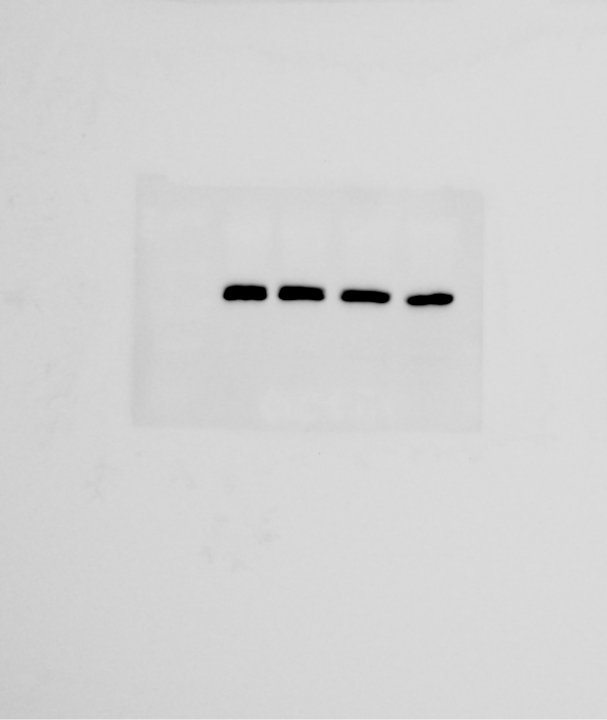

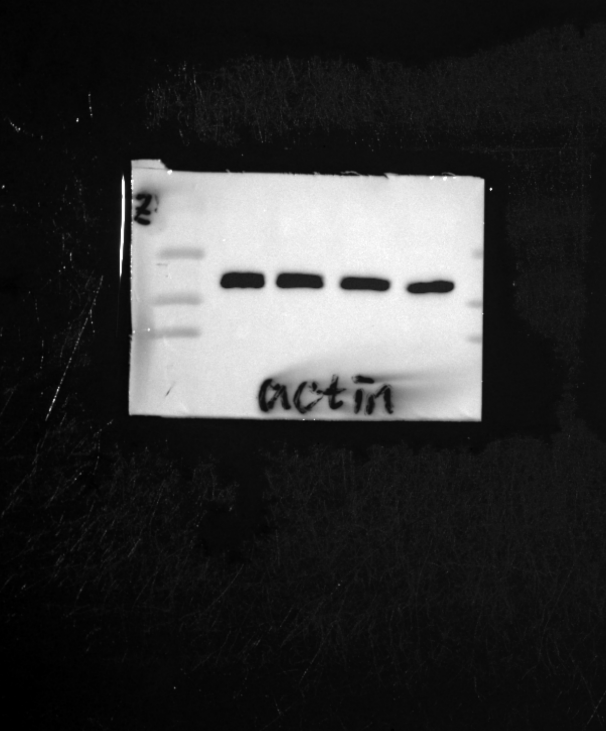


PTPN1


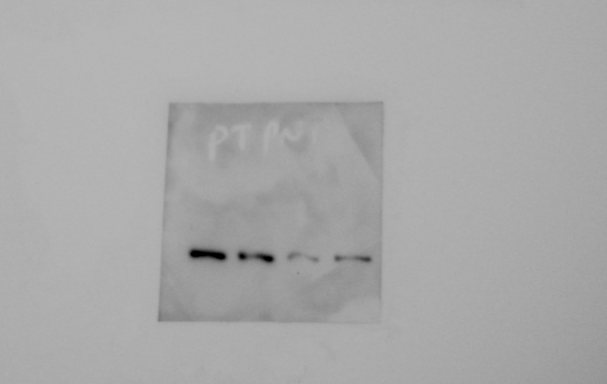

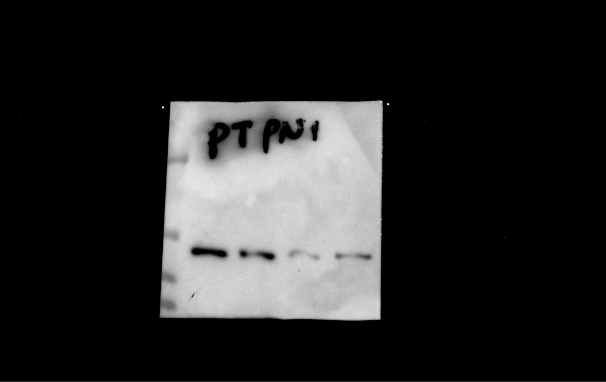


p‑IRS1


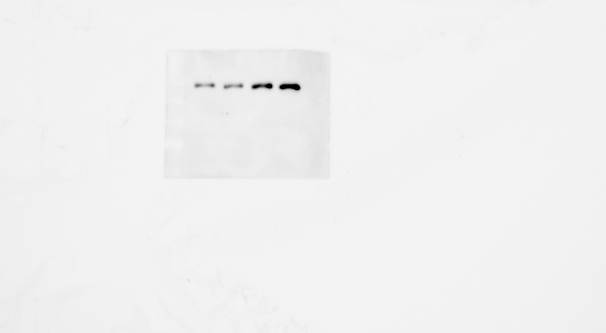

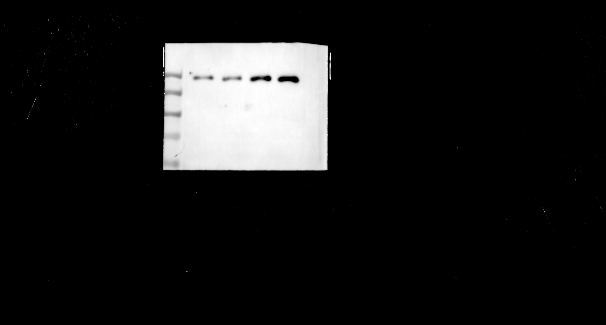


t‑IRS1


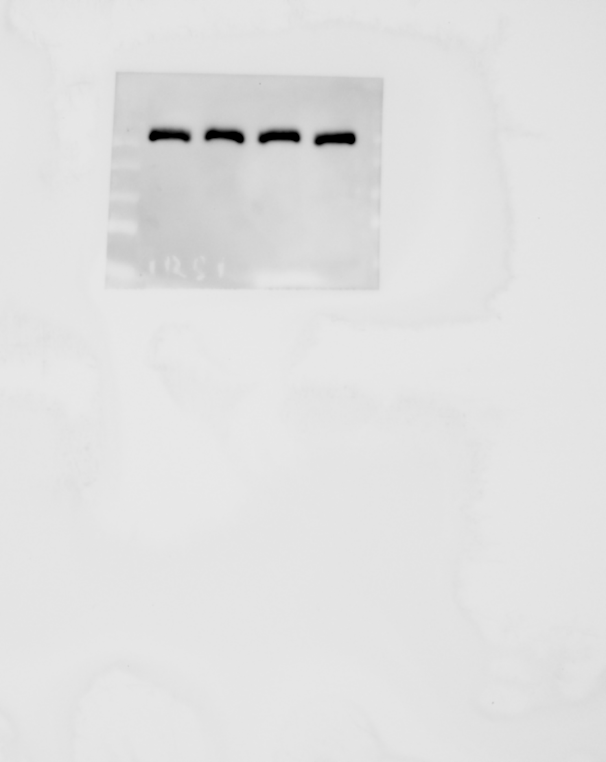

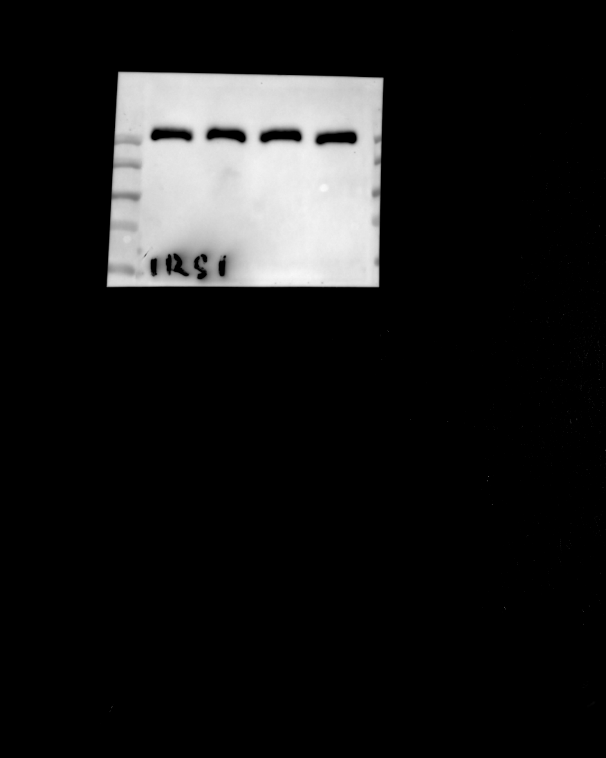


p‑AKT2


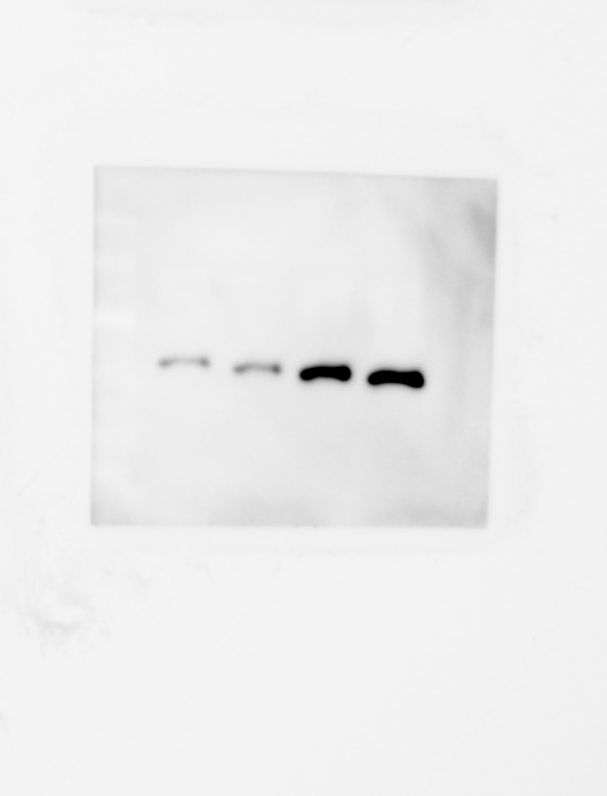

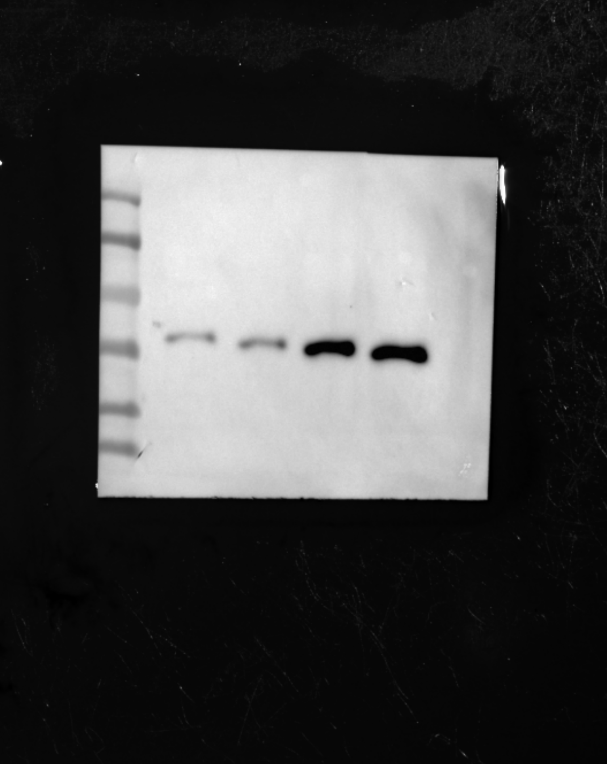


t‑AKT2


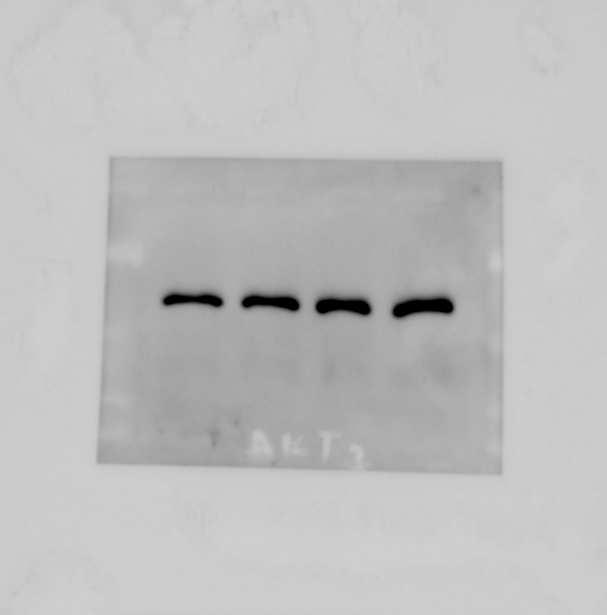

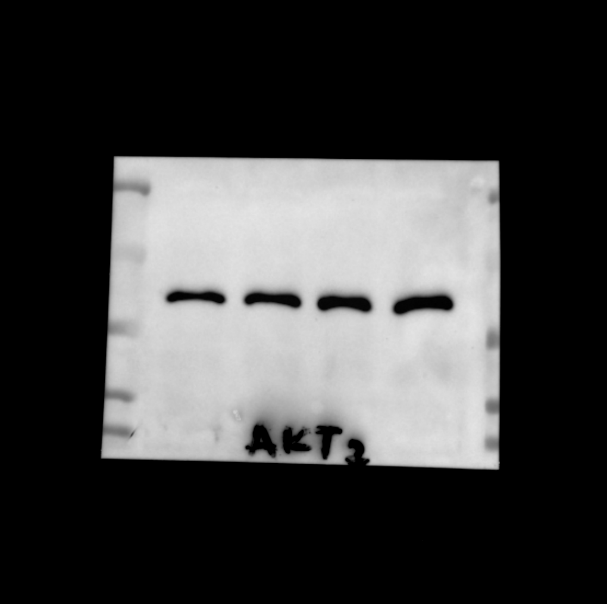


p‑mTOR


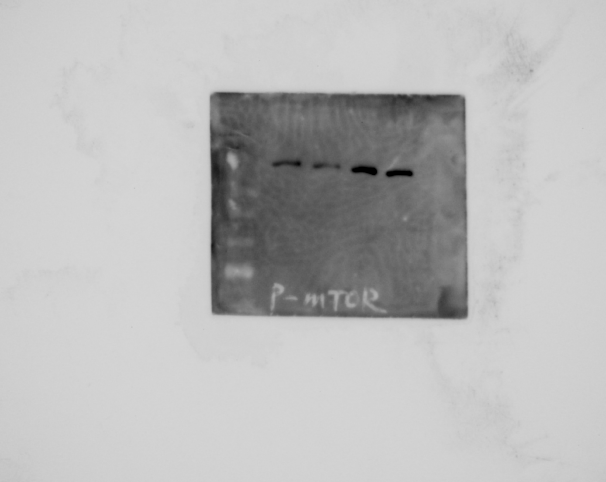

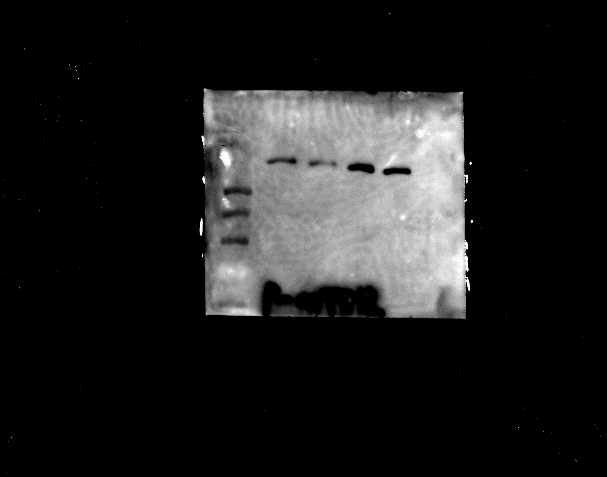


t‑mTOR


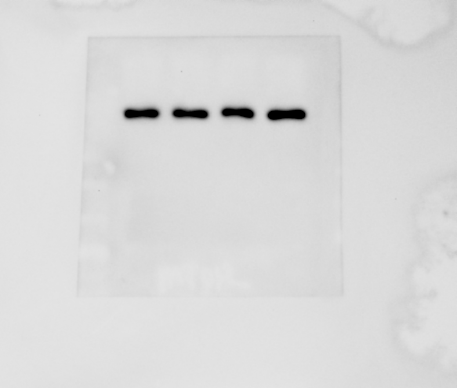

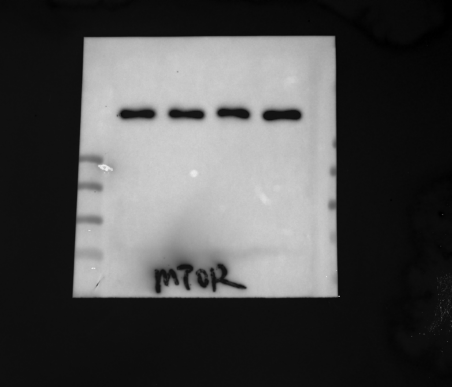


GLUT4


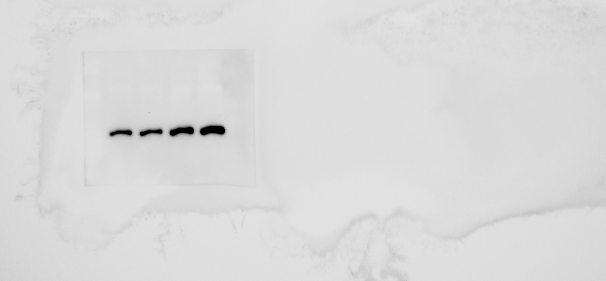

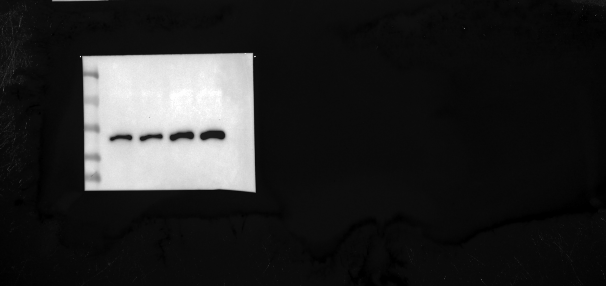


Figure 5H

β-actin


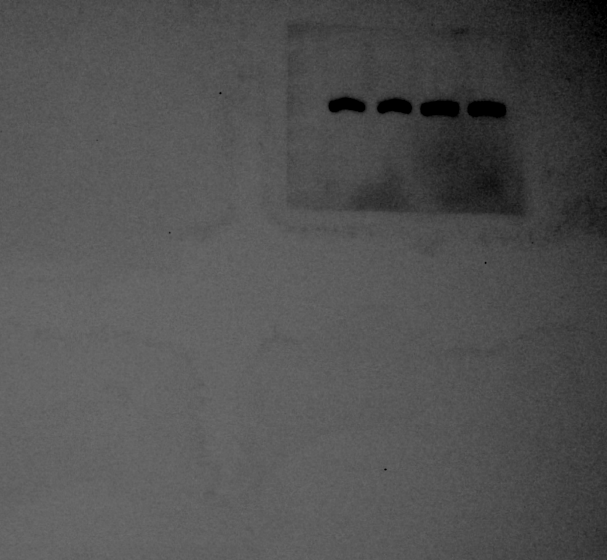

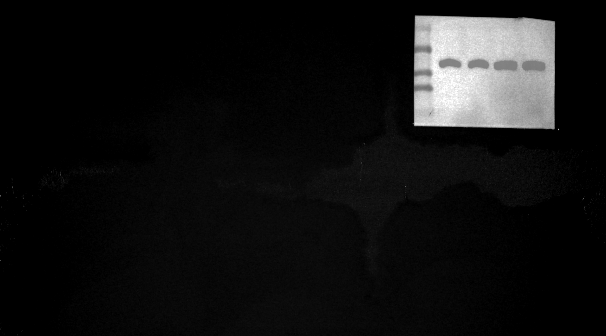


p‑IRS1


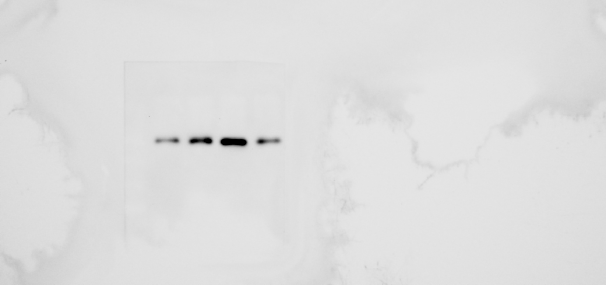

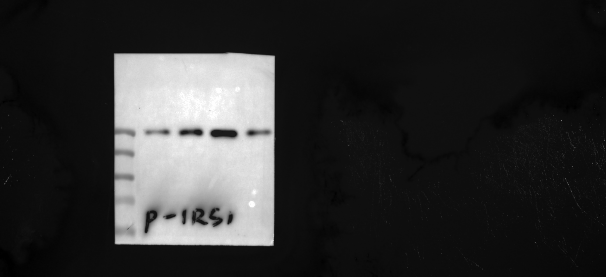


t‑IRS1


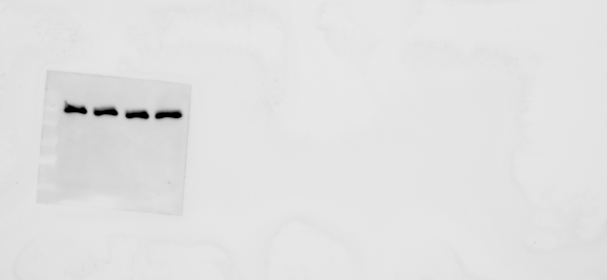

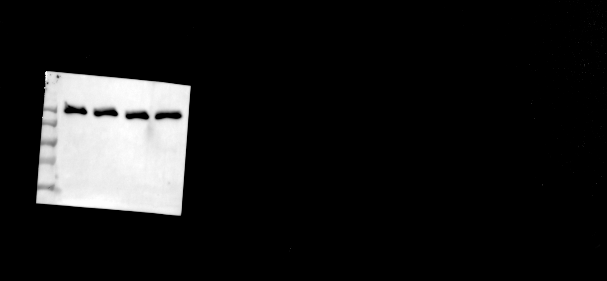


p‑AKT2


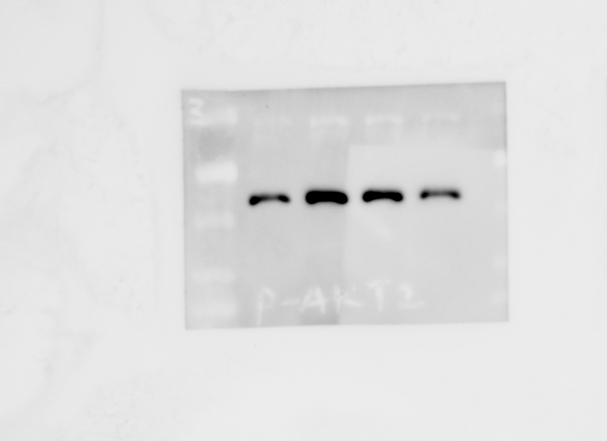

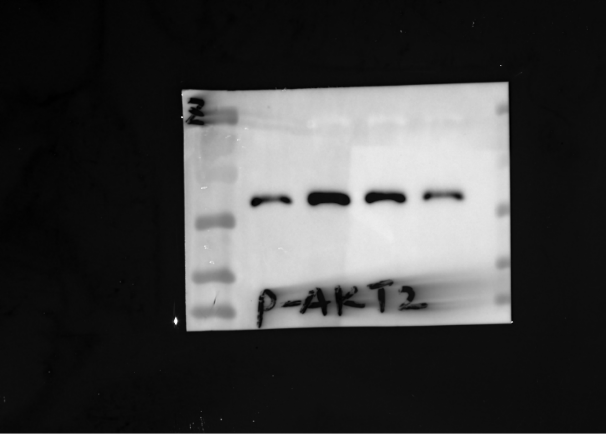


t‑AKT2


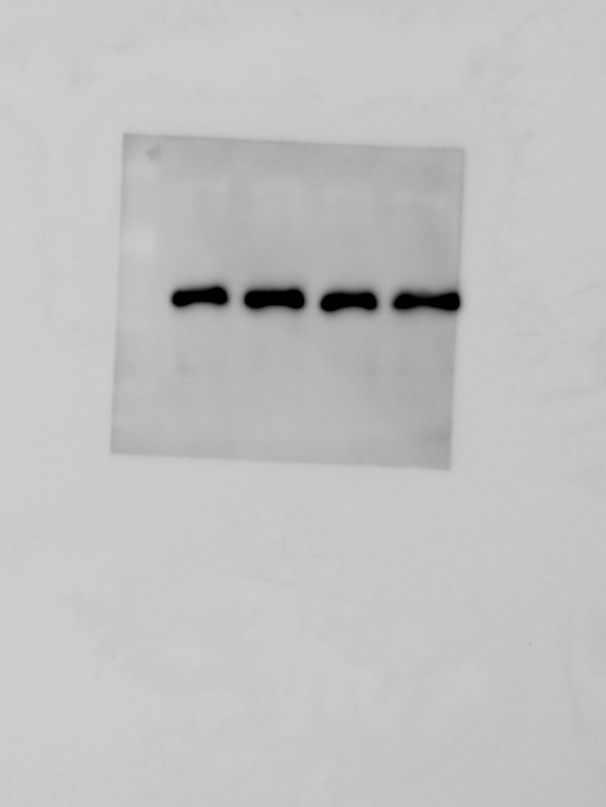

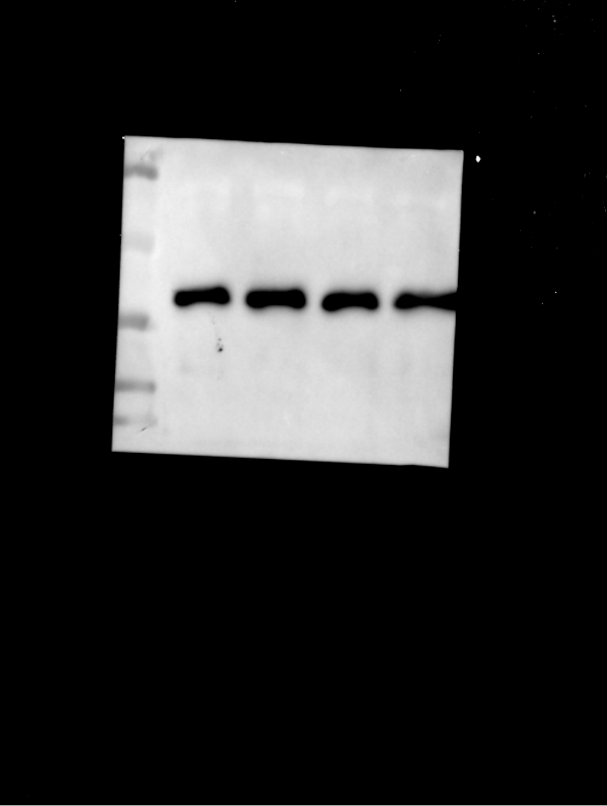


p‑mTOR


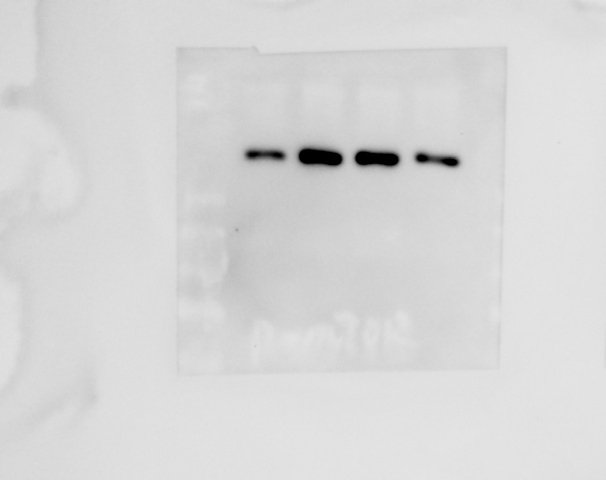

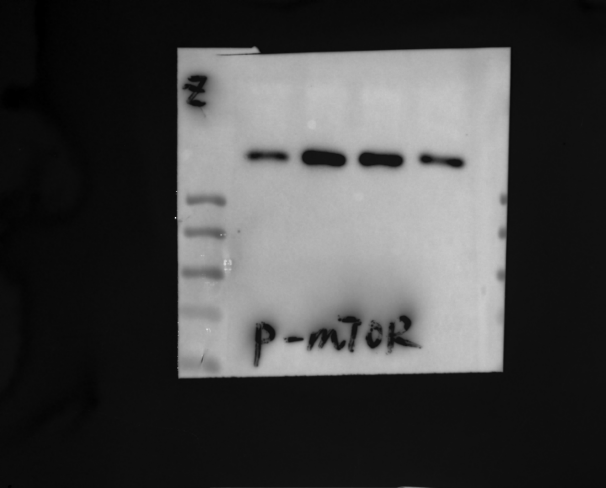


t‑mTOR


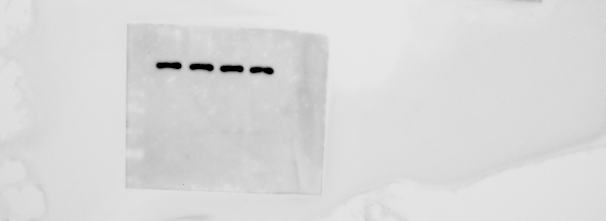

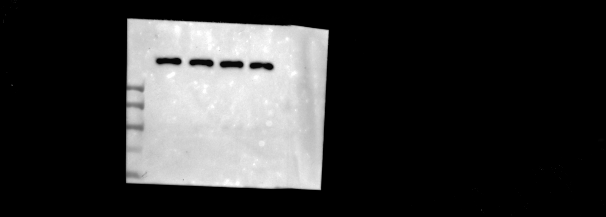


GLUT4


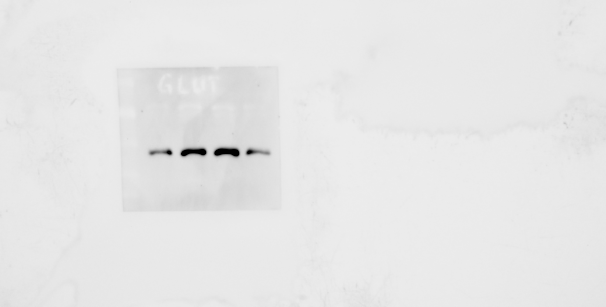

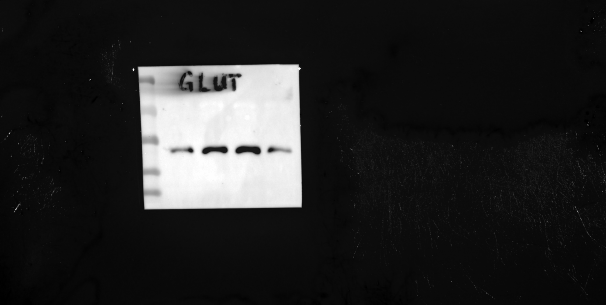


PTPN1


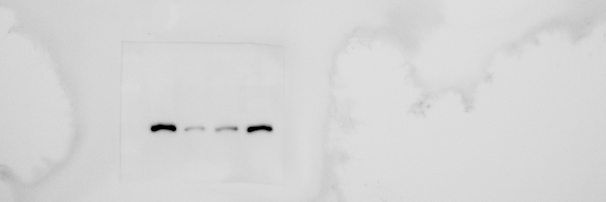

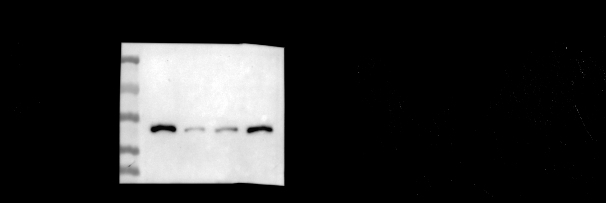


Figure 6

Figure 6B

β-actin


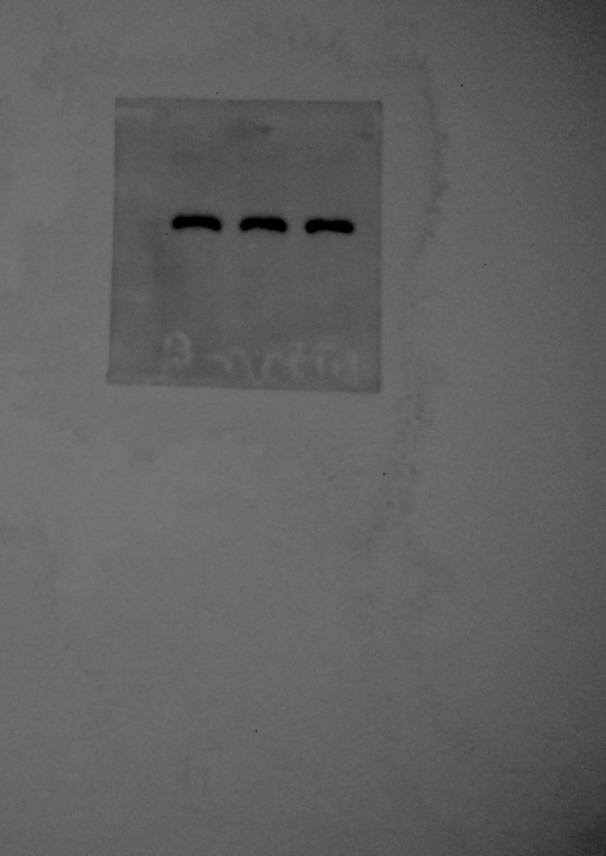

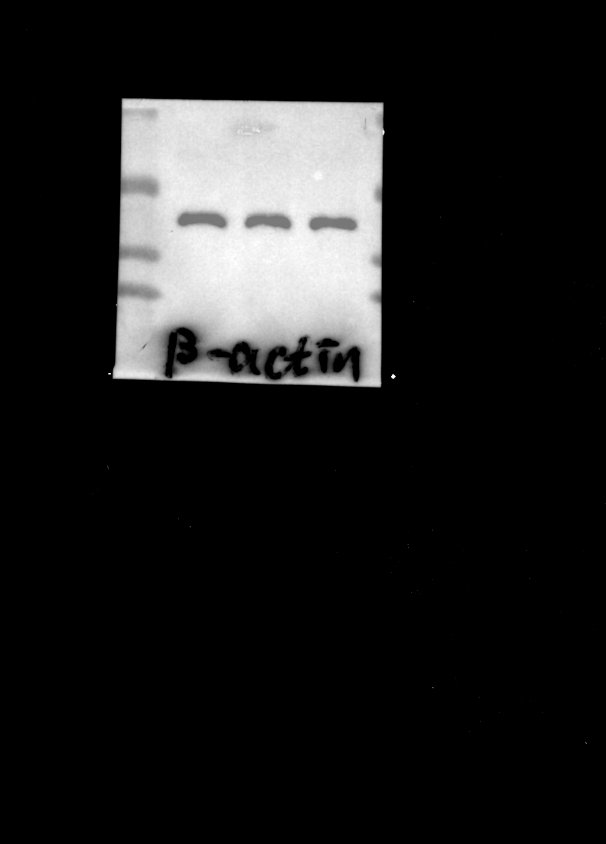


p-IRS1


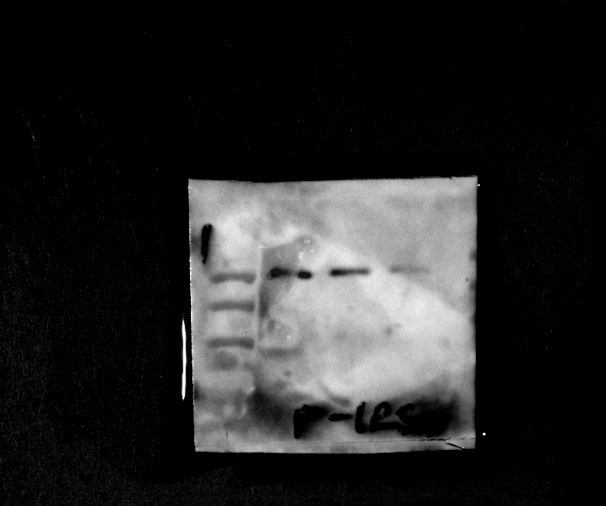

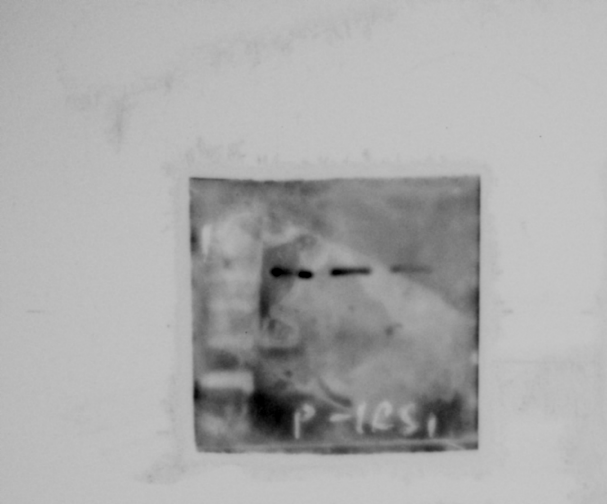


t-IRS1


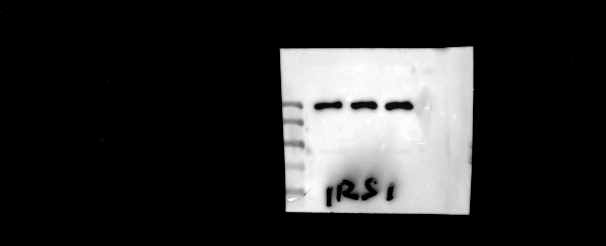

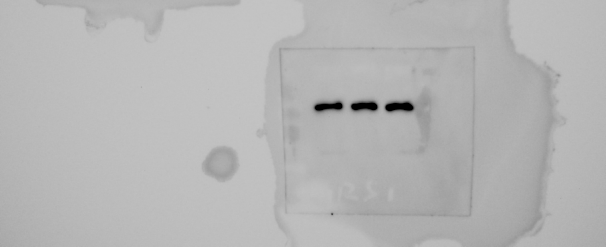


p-AKT2


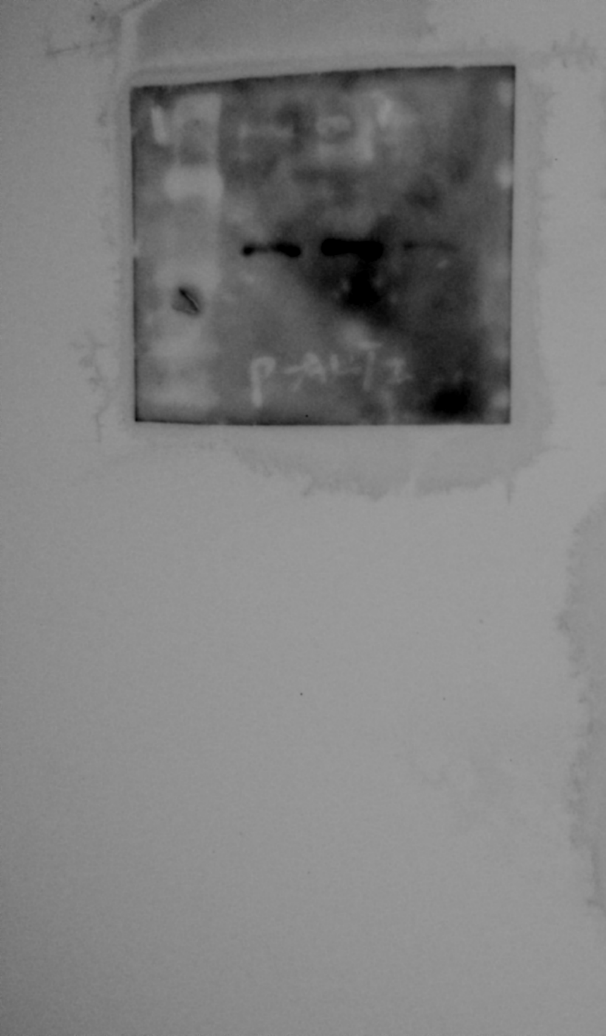

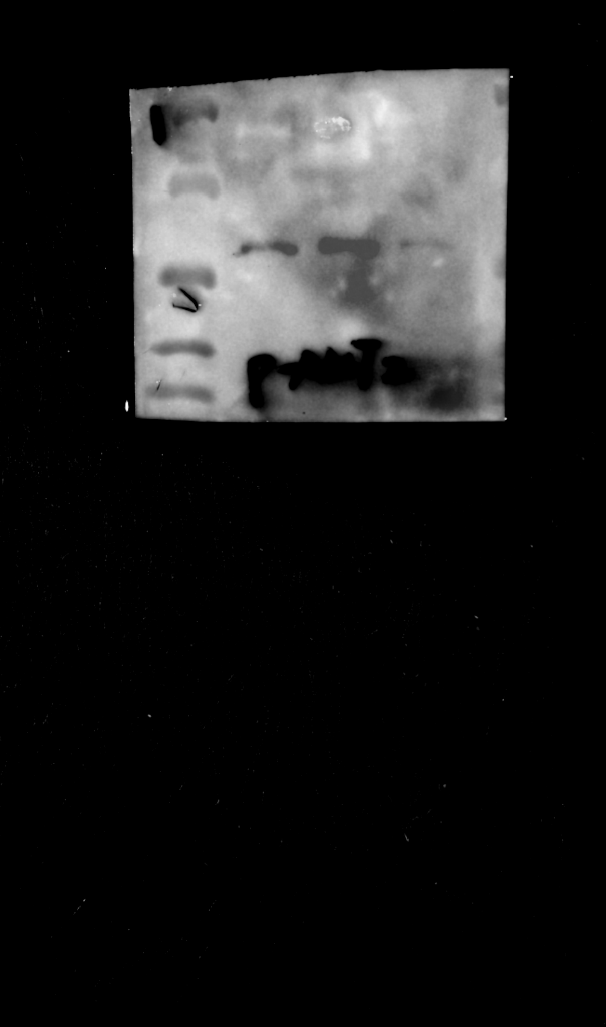


t-AKT2


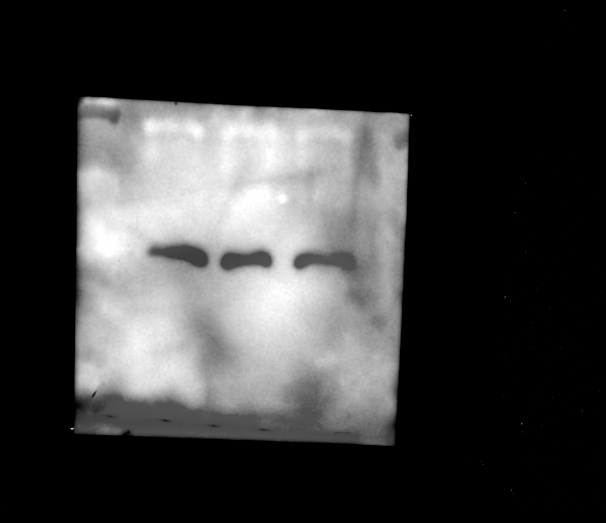

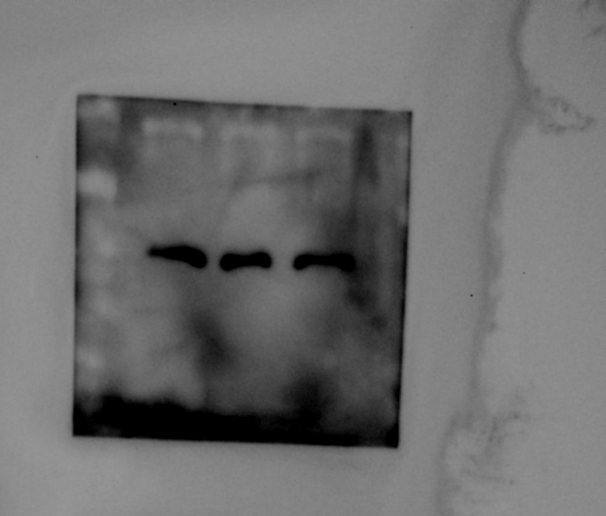


p-mTOR


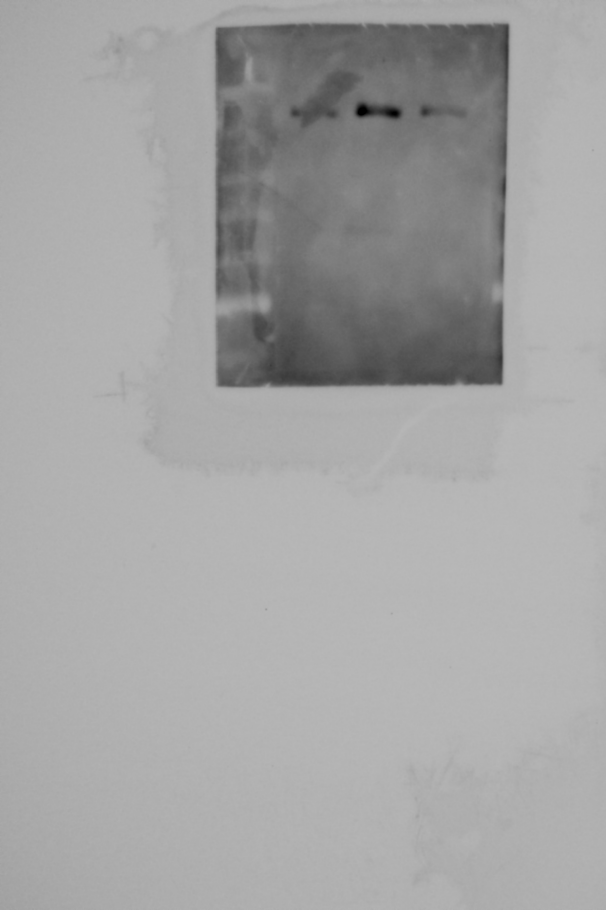

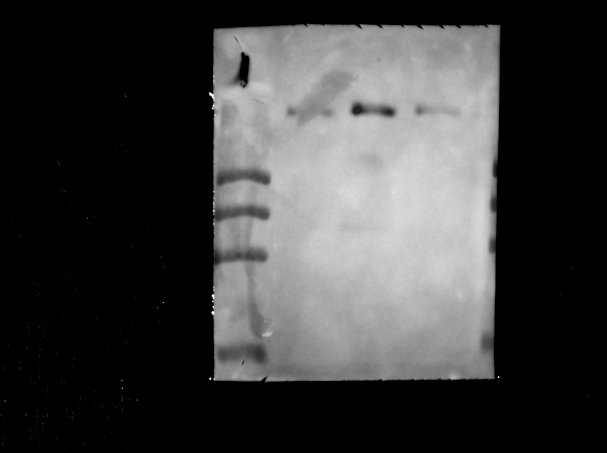


t-mTOR


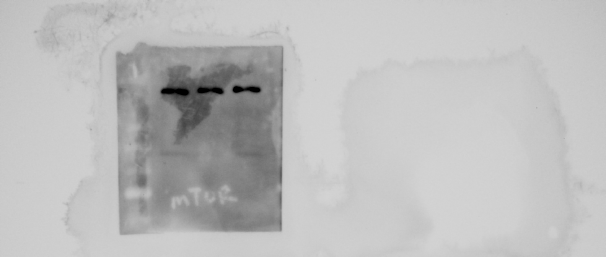

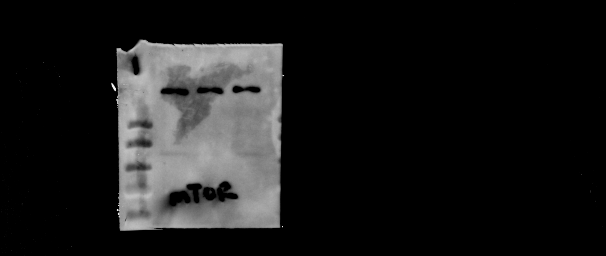


GLUT4


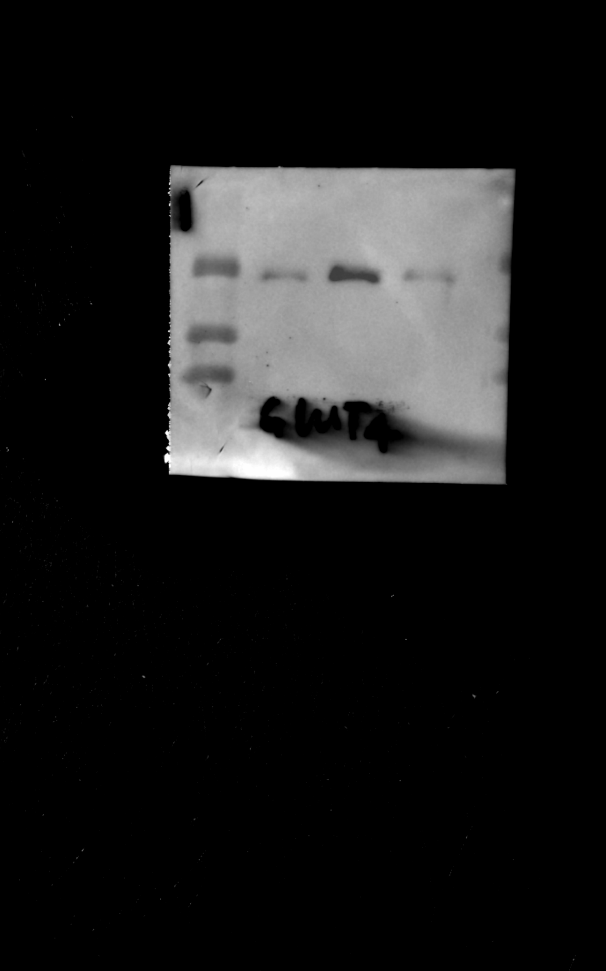

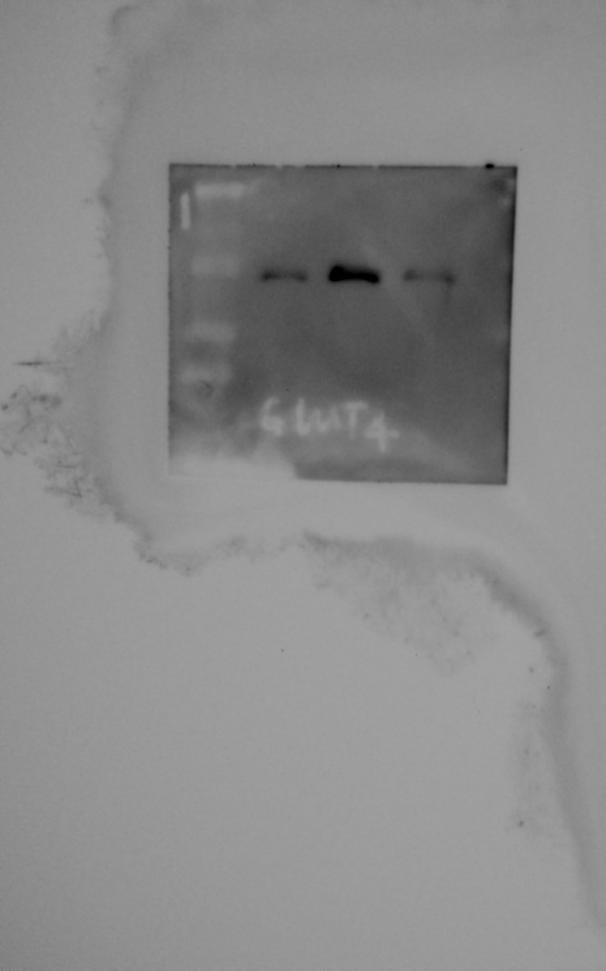


Supplementary Figure S5

Figure S5C

β-actin


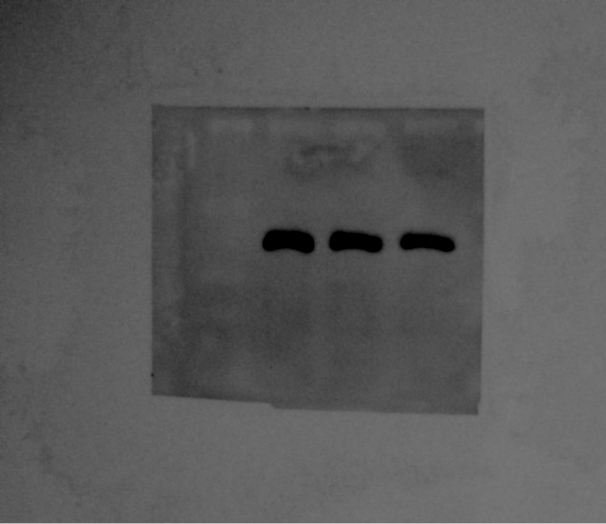

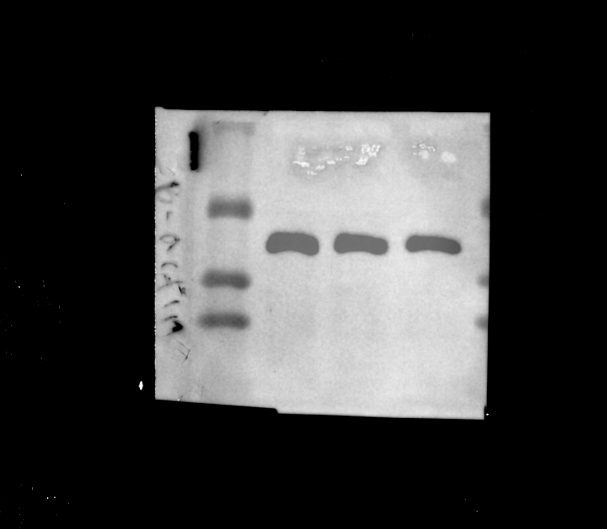


p-IRS1


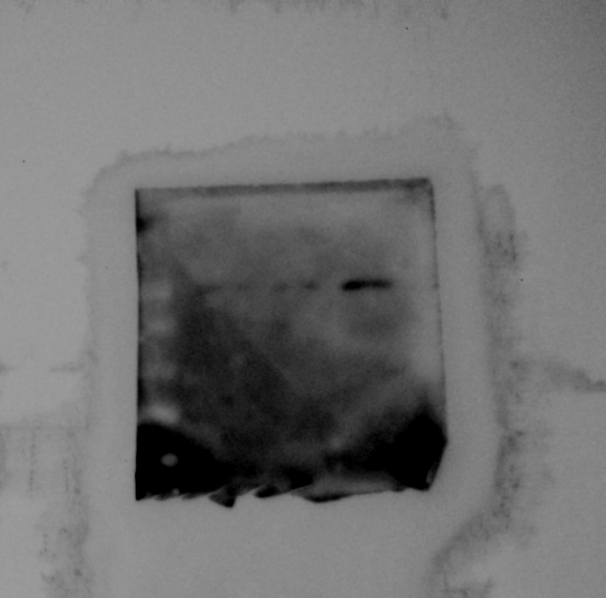

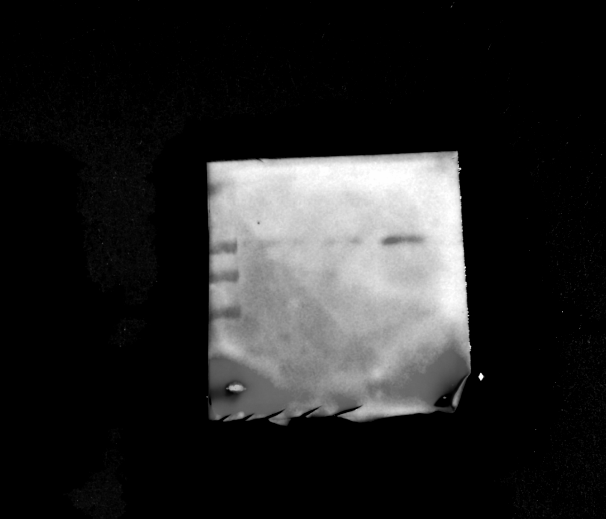


t-IRS1


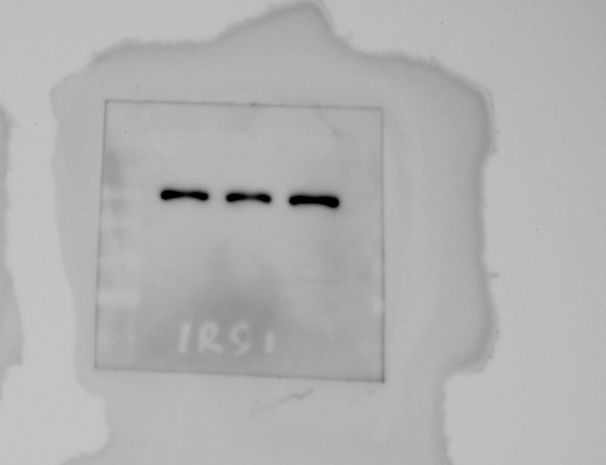

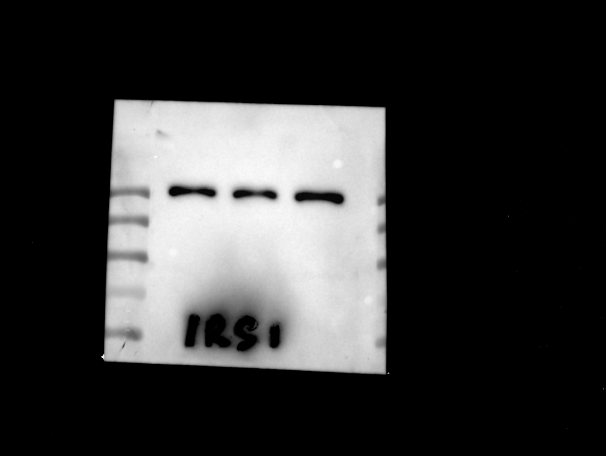


p-AKT2


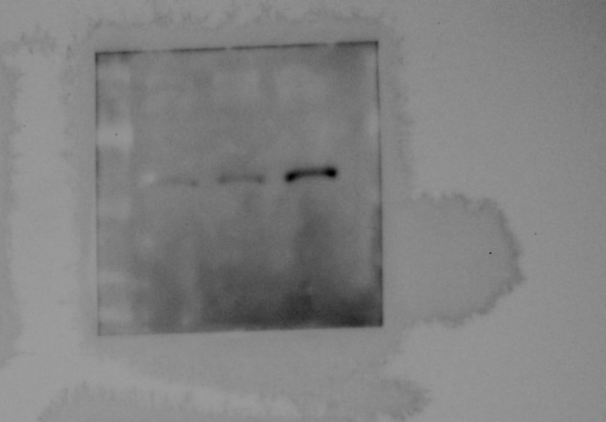

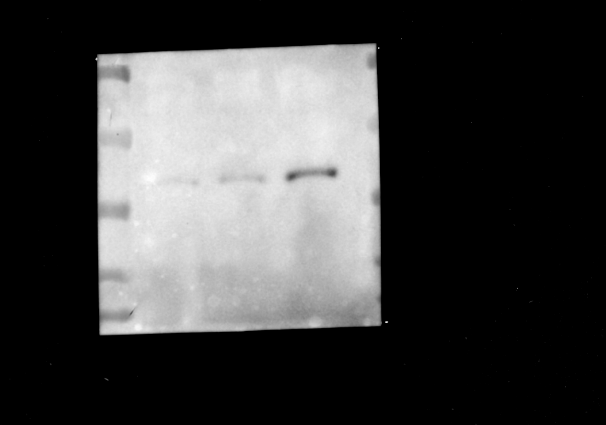


t-AKT2

p-mTOR

t-mTOR

GLUT4
